# CRISPR/Cas9 screen identifies DCAF4 as a novel protector of hepatocellular carcinoma against brachytherapy via stress granule-dependent NRF2 activation

**DOI:** 10.1038/s41419-026-09095-0

**Published:** 2026-07-14

**Authors:** Tian Huang, Yanshu He, XinChen Wang, Zhengan Liu, Enqi Qiao, Tong Sun, Junhao Mei, Shipeng Dai, Zhongkai Wang, Cheng Feng, Hengsong Cao, Yiming Wang, Yongxiang Xia, Enyu Liu, Jinhe Guo, Jian Lu

**Affiliations:** 1 Center of Interventional Radiology and Vascular Surgery, Basic Medicine Research and Innovation Center of Ministry of Education, Department of Radiology, Zhongda Hospital, School of Medicine, Southeast University, Nanjing, China; 2 Hepatobiliary Center, The First Affiliated Hospital of Nanjing Medical University, Key Laboratory of Liver Transplantation, Chinese Academy of Medical Sciences, NHC Key Laboratory of Living Donor Liver Transplantation, Nanjing, China; 3 Peking Union Medical College Hospital, Department of Liver Surgery, Peking Union Medical College Hospital, Chinese Academy of Medical Sciences and Peking Union Medical College (CAMS & PUMC), Beijing, China; 4 Organ Transplant Department, Qilu Hospital, Cheeloo College of Medicine, Shandong University, Jinan, China

**Keywords:** Radiotherapy, Apoptosis, Mechanisms of disease

## Abstract

Hepatocellular carcinoma (HCC) cells sustain viability and radioresistance by actively countering oxidative stress. Understanding the mechanisms regulating reactive oxygen species (ROS) homeostasis is therefore crucial for developing novel therapies. Using integrated genome-wide CRISPR-Cas9 screening coupled with transcriptomic and metabolomic profiling, we identified DDB1 and CUL4-associated factor 4 (DCAF4) as an essential regulator of oxidative stress resistance in HCC. Mechanistically, DCAF4 functions as a CRL4 E3 ligase adapter that promotes KEAP1 ubiquitination and degradation. Notably, under oxidative stress, cytoplasmic stress granules (SGs) form a localized platform that facilitates the DCAF4–KEAP1 interaction, accelerating KEAP1 degradation and leading to NRF2 activation and upregulation of antioxidant genes. We further identified that the transcription factor XBP1 enhances DCAF4 expression. Targeting this axis, we performed computational screening to identify a small-molecule inhibitor that disrupts the DCAF4–KEAP1 interaction. This compound effectively enhanced brachytherapy (BT) sensitivity and inhibited tumor growth in preclinical HCC models.

## Introduction

In 2022, there were approximately 866,000 new cases of liver cancer globally, with an incidence rate of 8.6 per 100,000, ranking eighth among all malignancies. Deaths from liver cancer reached about 759,000, with a mortality rate of 7.4 per 100,000, making it the fourth leading cause of cancer-related mortality worldwide. In China, primary liver cancer accounted for 367,700 new cases and 316,500 deaths annually, ranking fifth in incidence and second in mortality among all malignant tumors [1]. HCC represents the most common pathological type of primary liver cancer, comprising ~90% of all cases. Only about 30% of newly diagnosed HCC patients in China are eligible for surgical resection [2]. Brachytherapy (BT) is a potent therapeutic technique developed in recent decades, increasingly employed in the treatment of HCC owing to its exceptional ability to target and destroy cancer cells while sparing normal tissue [3]. Radiotherapy exploits ROS-induced oxidative stress to kill tumor cells, an effect counteracted by tumor antioxidant defense mechanisms [4]. Unlike conventional external beam radiotherapy, radioactive seed implantation delivers continuous low-dose-rate BT, offering advantages such as high conformality and sustained radiation exposure [5]. Therefore, the molecular mechanisms that govern the radioresistance of HCC urgently need to be explored.

The development and progression of HCC, which is often driven by chronic inflammatory conditions, is associated with disrupted redox homeostasis and ROS accumulation [6]. To identify key genes enabling cancer cell survival under such oxidative stress, genome-wide CRISPR/Cas9 screening has emerged as a pivotal functional genomics approach [7]. Applying this approach, we identified DCAF4 as essential for HCC cell survival under oxidative stress conditions. DCAF4 is a WD repeat-containing protein that acts as a substrate recognition adapter within the Cullin-RING ubiquitin ligase 4 complex. By interacting with DDB1, it facilitates the assembly of the E3 ubiquitin ligase complex, which recognizes specific substrates and mediates their ubiquitination, thereby influencing the stability, activity, or localization of target proteins [8]. Previous studies have shown that DCAF4 promotes autophagic degradation of the antioxidant enzyme SOD1 by mediating OPTN ubiquitination [9]. In colorectal cancer, DCAF4 specifically ubiquitinates the tumor suppressor ST7, thereby driving tumor initiation and progression [10]. Despite established roles for epigenetic and signaling pathways in radiosensitivity, the contribution of DCAF4 to HCC biology and internal radiotherapy response remains largely unknown.

Our latest findings demonstrate that the E3 ubiquitin ligase adapter DCAF4 promotes the ubiquitination and degradation of KEAP1 and influences its localization to SGs. This disruption leads to the release and nuclear translocation of NRF2, which in turn activates the transcription of antioxidant genes such as HO-1 and NQO1, enhancing cellular resistance to BT. Using virtual screening, we have identified for the first time that Rebaudioside M acts as a selective and cell-active DCAF4 inhibitor. To our knowledge, this compound represents a novel class of DCAF4 inhibitor and shows promise as a potential small-molecule therapeutic agent for mitigating oxidative stress in HCC.

## Materials and methods

### Samples collection

From 2014 to 2021, a total of 98 patients with HCC were managed at the First Affiliated Hospital of Nanjing Medical University. The cohort consisted of individuals aged between 30 and 83 years (mean age: 59.5 years), all of whom underwent initial curative resection with paired HCC tissues and adjacent non-tumorous tissues collected. Exclusion criteria included prior receipt of radiotherapy or chemotherapy, as well as incomplete clinicopathological records. HCC diagnoses were histopathologically confirmed by two independent pathologists. Overall survival was defined as the interval from the date of primary surgery to either death from any cause or the last follow-up visit.

### Metabolomics sequencing and detection

A solvent mixture of methanol, acetonitrile, and water (2:2:1) was prepared and chilled on ice. Subsequently, 1 mL of the precooled mixture was added to the samples, followed by sonication in an ice-water bath for 60 min. After incubation at −20 °C for 1 h, the samples were centrifuged at 14,000×*g* for 20 min at 4 °C. The resulting supernatants were collected for LC–MS analysis. Metabolomic profiling was performed using a UPLC-ESI-Q-Orbitrap-MS system, consisting of a Shimadzu Nexera X2 LC-30AD UHPLC coupled to a Q-Exactive Plus mass spectrometer (Thermo Scientific). Data were acquired in both positive and negative electrospray ionization (ESI) modes. For quality control, a pooled QC sample was prepared by combining equal aliquots of all samples and used for data normalization. Both QC and blank samples (75% acetonitrile in water) were injected every six experimental samples.

### Metabolome sequencing analysis

MS data were processed using MS-DIAL, with metabolite identification performed by matching MS/MS spectra against public databases and an in-house standard library. Permutation tests were applied to validate all multivariate models. Orthogonal projections to latent structures-discriminant analysis (OPLS-DA) was used to identify significant metabolites based on variable importance in projection (VIP) scores. Metabolites with VIP > 1 were considered relevant, with higher scores indicating greater contribution to group separation. For univariate analysis, statistical significance was assessed using two-tailed Student’s *t*-tests or ANOVA, with a *p*-value < 0.05 considered significant. Metabolites meeting both VIP > 1 and *p* < 0.05 were defined as differentially abundant. Cluster analysis of differential metabolites was performed using R. Functional interpretation was carried out via KEGG pathway enrichment analysis (http://www.kegg.jp). Enriched pathways with *p* < 0.05 were deemed statistically significant.

### Virtual screening and molecular docking

Potential active sites on the DCAF4 structure (AF-Q8WV16-F1) were predicted using AlphaFold. Subsequently, a structure-based virtual screening was performed against two commercial chemical libraries containing over 105,000 compounds. This screening utilized the grid-based ligand docking approach implemented in GLIDE (Schrödinger Maestro 12.8) to identify potential binding poses. The top five scoring compounds, sourced from MedChemExpress, were selected for further experimental validation.

To explore the binding modes between DCAF4 and KEAP1, molecular docking was conducted using HADDOCK. The protein structures of DCAF4 (AF-Q8WV16-F1) and KEAP1 (AF-Q14145-F1) were obtained from the AlphaFold database. The resulting docking complexes were visualized and analyzed with PyMOL 2.3.0 to characterize the key interaction patterns.

### Microscale thermophoresis (MST)

The interaction between Reb-M and DCAF4 was analyzed by microscale thermophoresis (MST) using a Monolith NT.115 instrument (NanoTemper Technologies). GFP-DCAF4 was expressed in HEK293T cells and lysed 48 h post-transfection. The lysate was diluted 1:1.5 with MST buffer (10 mM Na-phosphate, pH 7.4, 1 mM MgCl_2_, 3 mM KCl, 150 mM NaCl, 0.05% Tween-20) to optimize fluorescence and then titrated with Reb-M (0–1 mM; MedChemExpress). Lysate from untransfected HEK293T cells served as a background control. Measurements were performed in premium coated capillaries at 25 °C, using a 470 nm LED and full infrared laser power. Data were normalized and fitted using the Hill equation in MO Affinity Analysis v2.1.3 software.

### Molecular dynamics simulation

A 100-ns molecular dynamics (MD) simulation was conducted with GROMACS 2020.6 to compare the structural stability of wild-type and acetylated KEAP1. The protein, parameterized with the CHARMM36m force field, was solvated in a cubic box (1.0 nm margin) under periodic boundary conditions and neutralized with 0.15 mol/L NaCl. System preparation included energy minimization (steepest descent) and solvent/ion equilibration. The production simulation employed the leap-frog integrator at 303.15 K and 1 bar. Trajectories were analyzed after backbone alignment to calculate RMSD.

### Mass cytometry

Tumor tissue samples were collected from control and Reb-M@Lips-cRGD tumor-bearing C57BL/6J mice. The tissue was processed using the Miltenyi Mouse Tumor Isolation Kit (Miltenyi Biotec, Germany), which employs Percoll to remove debris and separate red blood cells. Further experimental details are described in our previous work [11].

### Statistical analysis

Data are presented as mean ± standard deviation (SD). All quantitative experiments were performed with at least three independent biological replicates. Statistical analyses were primarily conducted using GraphPad Prism 8.0, with a *p*-value ≤ 0.05 defined as statistically significant. Comparisons between groups were performed using Student’s *t*-tests or ANOVA. Overall survival was evaluated by the Kaplan–Meier method, and differences were assessed with the log-rank test. Associations between DCAF4 expression and clinicopathological features were examined using the *χ*² test.

Further details on “Materials and methods” are available in the supplementary materials.

## Results

### Integrated whole-genome CRISPR-Cas9 screen with database analysis to identify critical mediators of the antioxidant response

**Fig. 1:**
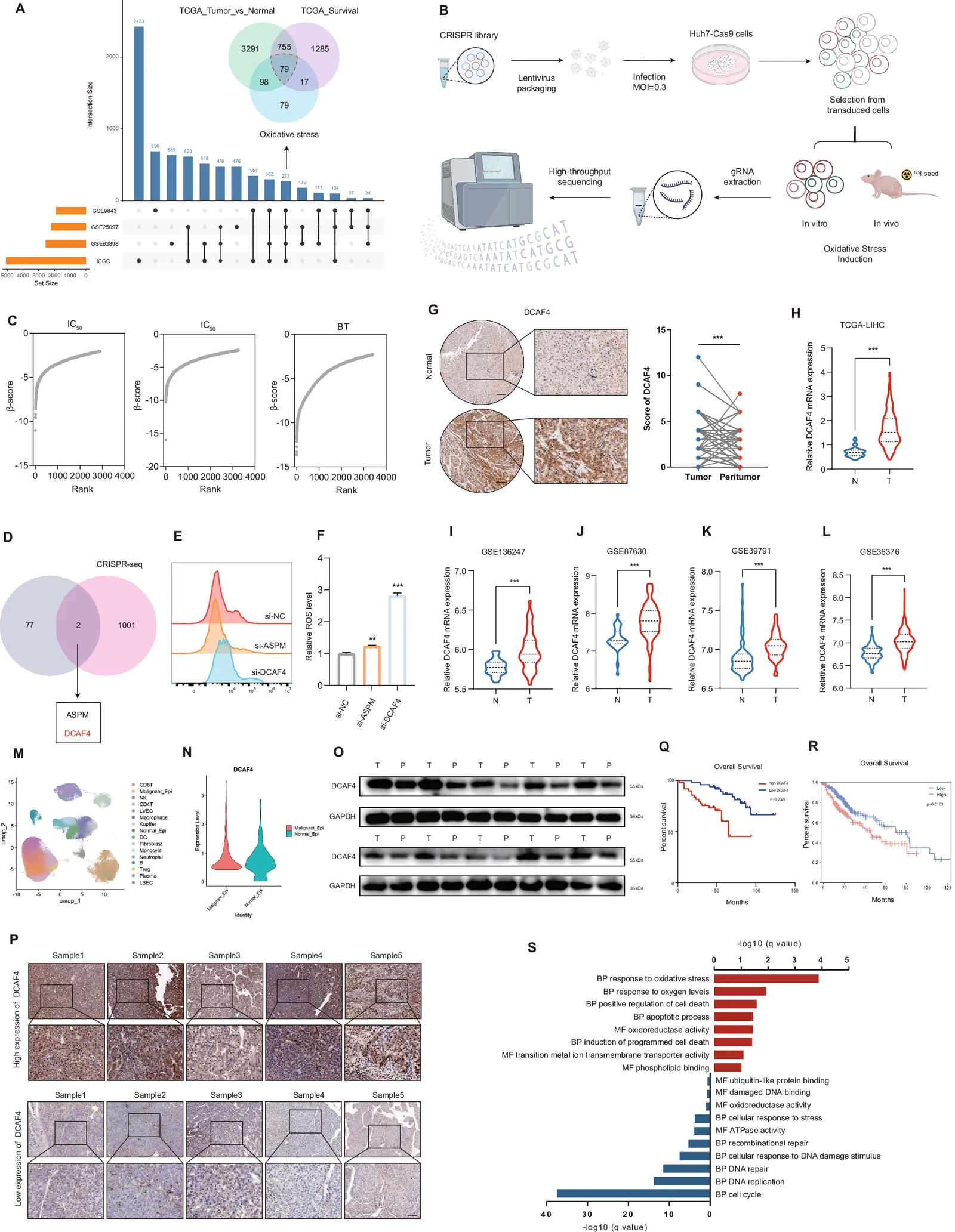
Genome-wide CRISPR screen identifies DCAF4 as a key regulator in HCC. **A** Oxidative stress scoring-based screening pipeline for HCC datasets. **B** Workflow of the genome-wide CRISPR screen in Huh7 cells to identify regulators of oxidative stress. **C** Normalized gene-level β-score for the top candidate genes identified in the CRISPR-Cas9 screen. **D** Venn diagram showing the intersection of genes from the CRISPR screen and oxidative stress scoring-based screening. **E** and **F** Flow cytometric analysis of intracellular ROS levels (left) and the corresponding median fluorescence intensity (MFI) (right) in Huh7 cells (*n* = 3). **G** Immunohistochemical staining depicting DCAF4 expression in HCC and matched adjacent normal tissues (scale bar, 200 μm). **H**–**L** Analysis of DCAF4 mRNA expression in HCC versus normal liver tissues from public datasets (TCGA, GSE136247, GSE87630G, GSE39791, and GSE36376). UMAP of single cells and DCAF4 expression; **M** UMAP of cell types in the integrated HCC scRNA-seq dataset; **N** violin plot of DCAF4 expression in malignant and normal epithelial cells. **O** Western blot analysis quantifying DCAF4 protein levels in paired HCC and adjacent normal tissues. **P** Representative IHC images showing high and low DCAF4 expression in HCC specimens (scale bar, 200 μm). **Q**, **R** Kaplan–Meier survival analysis of HCC patients based on DCAF4 expression was performed using a validation cohort (*n* = 98; HR = 3.474, 95% CI:1.514–7.974) and the TCGA database (HR = 1.59, 95% CI:1.11–2.27). *P*-values were determined by the log-rank test. **S** GO pathway enrichment analysis was conducted independently for the upregulated and downregulated DEGs following DCAF4 knockdown. *P < 0.05; **P < 0.01; ***P < 0.001.

To identify key genes that regulate oxidative stress in HCC and mediate BT sensitivity, we first applied the ssGSEA algorithm to calculate oxidative stress scores for HCC tissues in the GSE9843, GSE25097, GSE63898, and ICGC datasets. A total of 79 differentially expressed genes (DEGs) were identified across different datasets (Fig. 1A). Subsequently, we performed a genome-wide CRISPR-Cas9 screen in the HCC cell line Huh7 using the Brunello v2 library in vitro and in vivo to further delineate key genes, which comprises 77,441 gRNAs targeting 19,114 genes and 1000 non-targeting controls (Fig. 1B). First, we determined the dose–response curve of Huh7 cells to oxidative stress inducer TBHP treatment and established 48h IC_50_ and IC_90_ values of 28.46 and 110.2 μM, respectively (Fig. S1A). For the in vitro genetic screen, Cas9-expressing Huh7 cells were transduced with the sgRNA library and subsequently treated for 14 days with either vehicle control or TBHP at the IC_50_ or IC_90_ concentration. For the in vivo screen, oxidative stress was induced in HCC xenografts via ^125^I seed-BT over 14 days, as previously described [12, 13]. A baseline sample was collected after transduction and puromycin selection. sgRNAs that conferred either sensitivity or resistance to oxidative stress-induced cell death were identified via deep sequencing. We applied the Model-based Analysis of Genome-wide CRISPR-Cas9 Knockout (MAGeCK) algorithm to identify negatively selected genes under oxidative stress conditions. Greater variability was observed in the IC_50,_ IC_90_, or BT groups, likely indicative of stronger selection pressure (Fig. S1B). Genes were filtered based on β-score in each comparison (Figs. 1C and S1C). Ultimately, we identified 1003 overlapping negatively selected genes across different treatment groups, representing a core set of potential genes conferring resistance to oxidative stress in HCC (Fig. S1D). We integrated CRISPR screening data of negatively selected genes with above 79 DEGs. Venn analysis identified two candidate genes implicated in regulating oxidative stress response in HCC (Fig. 1D). To evaluate the impact of ASPM and DCAF4 on ROS levels in HCC, we performed individual knockdown of each gene using siRNA (Fig. S1E). Notably, knockdown of DCAF4 resulted in a significant increase in ROS levels in HCC cells (Fig. 1E and F).

To further assess the expression level of DCAF4, we applied the immunohistochemistry assay to 98 paraffin-embedded specimens obtained from HCC patients, revealing that HCC tissues indeed possess higher levels of DCAF4 protein than adjacent paracancerous tissues(Fig. 1G), a finding that echoes the results from the TCGA (Fig. 1H) and GEO (GSE87630, GSE136247, GSE39791, and GSE36376) (Fig. 1I–L). Analysis of an integrated single-cell dataset was performed to assess DCAF4 expression, contrasting malignant hepatic epithelial cells with their normal counterparts (Fig. 1M, N and S1F–K). Western blot was then performed on 10 paired HCC and para-cancerous tissues and showed that DCAF4 was upregulated in HCC tissues (Fig. 1O). Representative DCAF4 high or low expression immunohistochemistry is shown in Fig. 1P. Increased DCAF4 mRNA and protein expression levels in HCC cell lines compared to hepatocyte cell line THLE-2 (Fig. S1L and M). Clinical–pathological features suggested that DCAF4 expression has a significant correlation with tumor size, microvascular invasion, and Edmondson stage (Table S4). The Kaplan–Meier survival curve showed a reduced overall survival rate for patients with HCC who had high DCAF4 expression (Fig. 1Q and R). These findings highlight that DCAF4 is significantly upregulated in HCC, suggesting a poor prognosis for HCC patients.

To elucidate the mechanisms through which DCAF4 regulates oxidative stress in HCC, we performed RNA sequencing in control and DCAF4-knockdown Huh7 cells. Differentially expressed genes can be seen in the heat maps and volcano plots (Fig. S1N and O). Upon observing the si-DCAF4 group, it was found that a total of 700 mRNAs were significantly reduced, whereas 606 mRNAs showed considerable upregulation. KEGG analysis of the results revealed that a large proportion of the differentially downregulated genes were clustered in the p53 signaling pathway, apoptosis, and oxidative phosphorylation, as depicted in Fig. S1P. From the GO analysis, the most prevalent activities associated with these genes involve biological processes such as response to oxidative stress, as well as molecular functions including oxidoreductase activity and ubiquitin-like protein binding (Fig. 1S).

### DCAF4 protects HCC cells from BT-induced oxidative damage

In the initial phase of our investigation, we designed three shRNA constructs (designated sh1, sh2, and sh3) targeting DCAF4 for knockdown experiments in Huh7 and HCCLM3 cell lines. Comprehensive evaluation using both qRT-PCR and western blot analysis demonstrated that sh1 and sh2 mediated a substantial suppression of DCAF4 expression at the transcriptional and translational levels, as illustrated in Fig. S1Q–T. Additionally, we established a DCAF4-overexpressing Hep3B cell line via transfection with DCAF4-encoding plasmids (Fig. S1U and V).

**Fig. 2:**
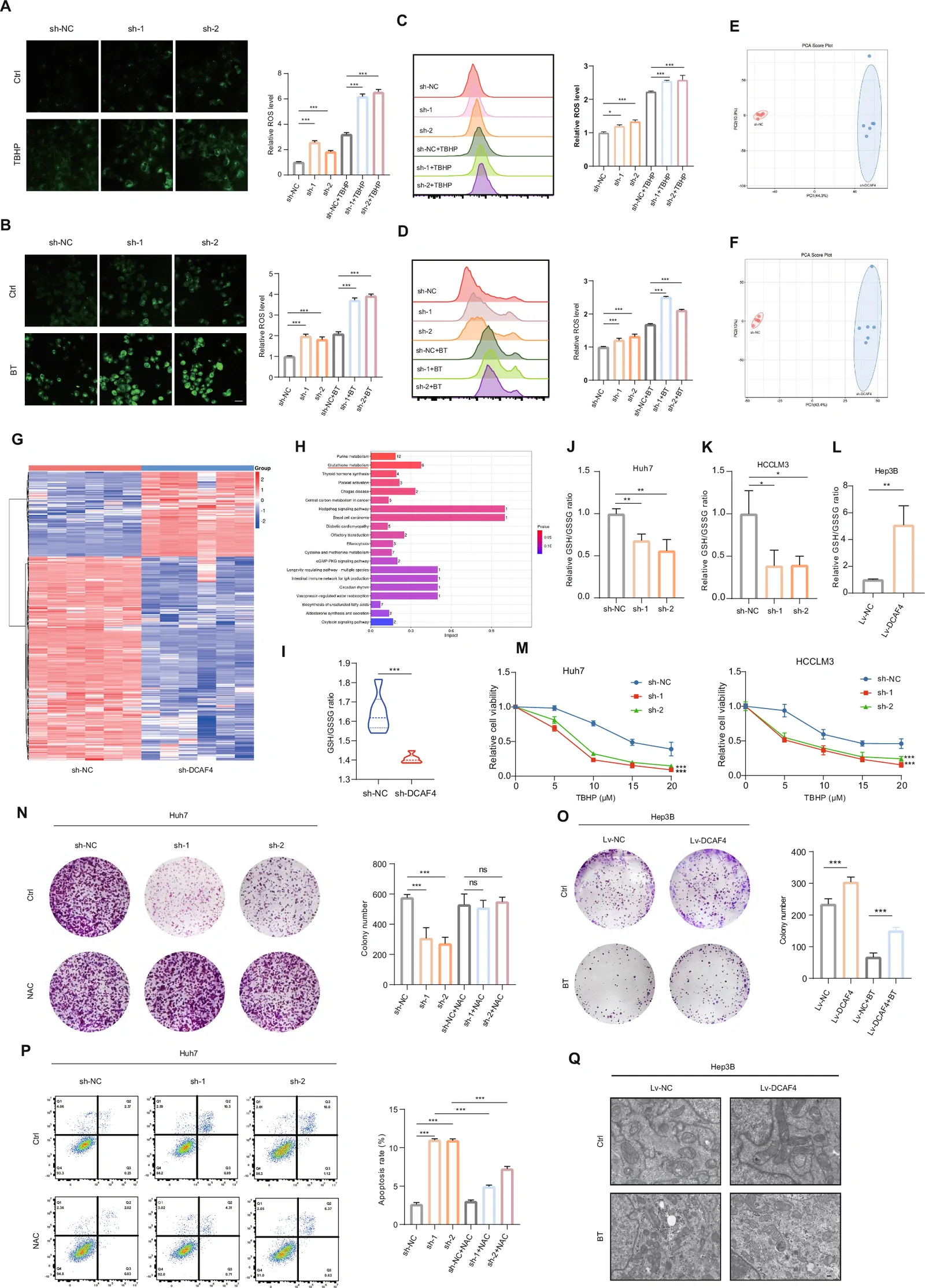
DCAF4 confers BT resistance in HCC. **A** and **B** Representative immunofluorescence images (left) and quantification (right) of HCC cells transfected with sh-NC, sh-1, or sh-2, showing intracellular ROS levels via DCFH-DA staining after 24 h treatment with 10 μM TBHP or 4 Gy BT (*n* = 3). Scale bars: 20 μm. **C** and **D** Flow cytometric analysis (left) and quantification (right) of Huh7 and HCCLM3 cells transfected with sh-NC, sh-1, or sh-2, showing intracellular ROS levels via DCFH-DA staining after 24 h treatment with 10 μM TBHP or 4 Gy BT(*n* = 3). **E** and **F** PCA score plots generated in positive and negative ion modes. **G** Heatmap depicting significantly altered metabolites in HCC cells between sh-NC and sh-DCAF4 groups. **H** Top enriched pathways from integrated pathway analysis of significantly changed metabolites. **I** Metabolomics-based analysis of the GSH/GSSG ratio. **J**–**L** Relative GSH/GSSG ratios in Huh7 and HCCLM3 cells after transfection with sh-NC, sh-1, or sh-2, and in Hep3B cells after transfection with Lv-NC or Lv-DCAF4 (*n* = 3). (**M**) Viability of Huh7 and HCCLM3 cells transfected with sh-NC, sh-1, or sh-2, assessed by CCK-8 assay after 24 h TBHP treatment (*n* = 3). **N** and **O** Representative images (left) and quantification (right) of colony formation assays in DCAF4-silenced Huh7 cells treated with NAC (2 mM), or DCAF4-overexpressing Hep3B cells treated with BT (4 Gy)(*n* = 3). **P** Apoptosis in Huh7 cells transfected with sh-NC, sh-1, or sh-2 and treated with NAC (2 mM, 24 h), analyzed by flow cytometry using an Annexin V/PI Apoptosis Detection Kit (*n* = 3). **Q** Representative transmission electron microscopy (TEM) images illustrating mitochondrial morphology in Hep3B cells transfected with Lv‑NC or Lv‑DCAF4 and subsequently exposed to BT (4 Gy). Scale bars: 200 nm. *P < 0.05; **P < 0.01; ***P < 0.001.

To assess the potential involvement of DCAF4 in oxidative stress regulation, we measured intracellular ROS levels using the cell-permeable fluorescent probe DCFH-DA. Under both basal conditions and upon challenge with the oxidative stress inducer TBHP or BT, DCAF4 knockdown significantly augmented total ROS levels in HCC cells compared to control groups (Fig. 2A–D). Given the established context of bidirectional regulation between oxidative stress and tumor metabolism [14], we sought to characterize the metabolic function of DCAF4, a potential modulator within this network. Using non-targeted LC–MS metabolomics in DCAF4-knockdown Huh7 cells, we identified the specific metabolic alterations induced by DCAF4. The overall distribution trend among samples was visualized by principal component analysis (PCA) (Fig. 2E and F). As visualized by heatmap and volcano plot, DCAF4 knockdown induced a significant shift in the metabolomic profile, with glutathione thiol, tetaine, and flunixin meglumine being among the top 50 most significantly altered metabolites (Figs. 2G and S2A, B). Enrichment analysis of the differential metabolites identified glutathione metabolism as one of the most significantly perturbed pathways upon DCAF4 silencing (Figs. 2H and S2C). Glutathione (GSH), the central cellular antioxidant, orchestrates ROS homeostasis and related physiology, with profound implications for tumor progression and drug resistance [15]. Metabolomic profiling initially suggested that DCAF4 silencing perturbs the glutathione system (Fig. 2I). To directly assess the impact on cellular redox state, we quantified intracellular glutathione levels. Confirming this, DCAF4 depletion caused a marked decline in the GSH/GSSG ratio in both HCC cell lines, indicating compromised antioxidant capacity. Conversely, DCAF4 overexpression elevated the ratio (Fig. 2J–L). Moreover, lipid peroxidation levels were assessed via flow cytometry using the fluorescent indicator C11-BODIPY. As depicted in Fig. S2D–I, DCAF4 silencing led to a pronounced increase in lipid ROS, whereas DCAF4 overexpression significantly attenuated lipid peroxidation. These collective findings suggest that DCAF4 plays a promotive role in enhancing the antioxidative capacity of HCC cells.

Excessive production of ROS induces oxidative damage to various cellular components, including membrane lipids, proteins, and DNA, ultimately triggering cell death through multiple pathways such as ferroptosis, apoptosis, and PARthanatos [16]. CCK-8 assay demonstrated that under oxidative stress, knockdown of DCAF4 led to a significant reduction in cell viability in HCC cells compared to sh-NC controls, indicating enhanced susceptibility to oxidative stress (Fig. 2M). The suppression of colony formation caused by DCAF4 inhibition could be effectively rescued by treatment with N-Acetylcysteine (NAC), a ROS inhibitor. Overexpression of DCAF4 attenuated BT-induced suppression of colony formation (Fig. 2N and O). Annexin V/PI staining further revealed that DCAF4 knockdown increased the proportion of apoptotic and necrotic cells relative to sh-NC controls. The enhanced apoptosis caused by DCAF4 inhibition could be effectively rescued by treatment with NAC (Figs. 2P and S2J, K). In contrast, overexpression of DCAF4 reduced BT-induced apoptosis (Fig. S2L and M). Moreover, transmission electron microscopy (TEM) analysis indicated that Lv-NC Hep3B cells exhibited more severe mitochondrial shrinkage and higher membrane density compared to Lv-DCAF4-expressing cells after BT (Fig. 2Q). In summary, these collective findings suggest that DCAF4 influences BT sensitivity by modulating ROS levels and redox homeostasis.

### DCAF4 drives HCC tumor growth in vivo by directly attenuating intracellular ROS levels

**Fig. 3:**
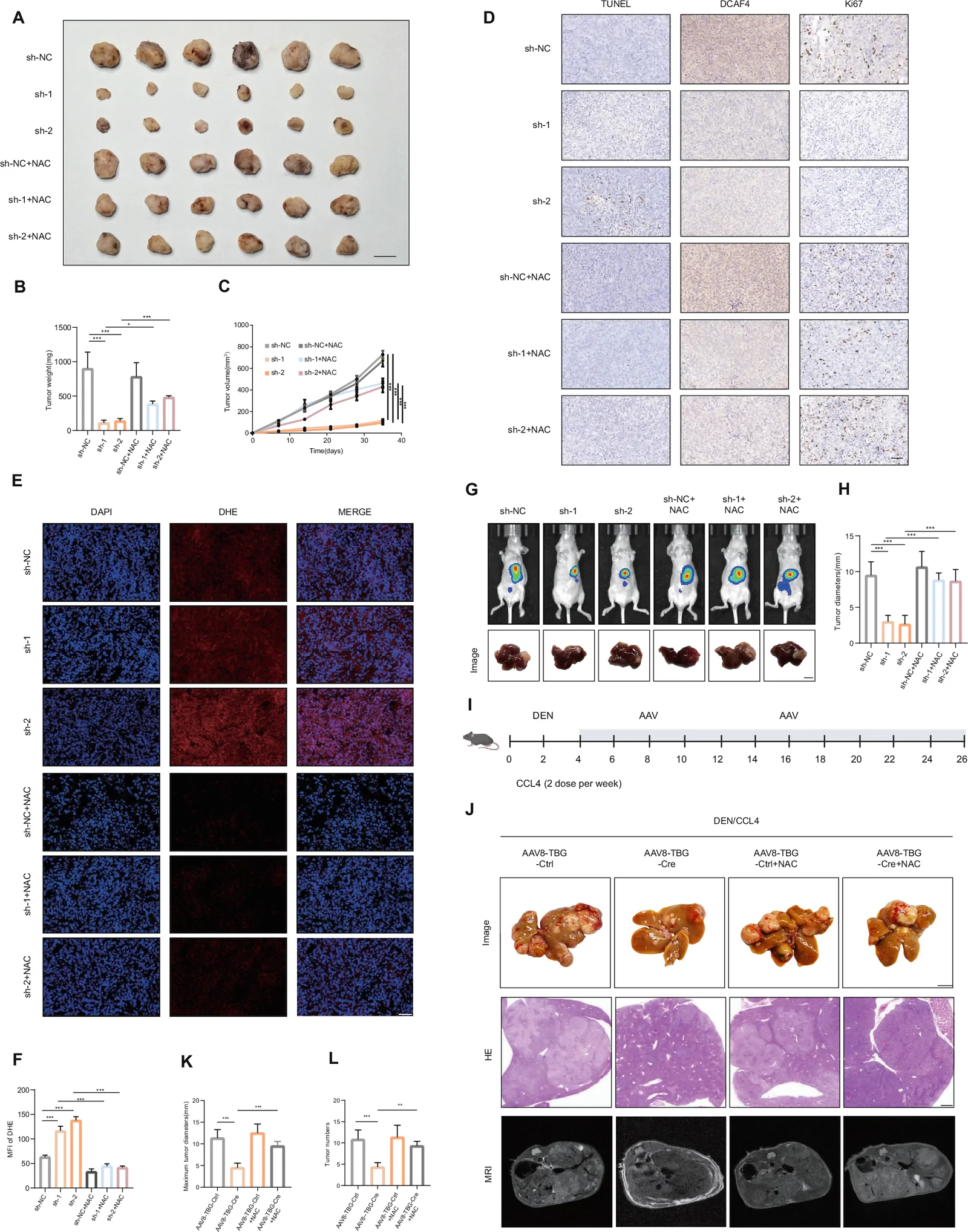
DCAF4 drives antioxidant defense in HCC in vivo. **A** Nude mice injected with DCAF4-depleted or control Huh7 cells were administered distilled water (Ctrl) or NAC (120 mg/kg/day), followed by a tumor formation assay (*n* = 6). Scale bars: 1 cm. **B** and **C** Tumor growth and weight were statistically analyzed in the subcutaneous xenograft model. **D** IHC staining of xenograft tissues for TUNEL, DCAF4, and Ki67. Scale bars: 50 μm. **E** and **F** Representative immunofluorescence images and quantitative analysis of DHE levels in each group. Scale bars: 50 μm. **G** and **H** Bioluminescence images of mice injected with DCAF4-depleted or control Huh7 cells and treated with distilled water (Ctrl) or NAC (120 mg/kg/day), acquired at the experimental endpoint. Tumor diameters are shown on the right. Scale bars: 5 mm. **I** Schematic of the in vivo strategy to evaluate the effect of AAV8-TBG-Cre on tumor development in a DEN/CCl_4_-induced HCC model. **J**–**L** Representative macroscopic images (scale bars: 5 mm), H&E staining (scale bars: 1 mm), and microMRI of livers from AAV8-TBG-Ctrl and AAV8-TBG-Cre-treated mice administered distilled water (Ctrl) or NAC (120 mg/kg/day). Tumor numbers and diameters are shown on the left. *P < 0.05; **P < 0.01; ***P < 0.001.

Our previously conducted in vitro experiments demonstrated that DCAF4 enhances the proliferative capacity of HCC by modulating redox homeostasis. To further determine whether DCAF4 attenuates intracellular ROS levels to facilitate tumorigenesis in vivo, we established a subcutaneous xenograft mouse model. Knockdown of DCAF4 markedly impaired tumor growth, as evidenced by reduced tumor size and weight, whereas administration of the antioxidant NAC restored tumorigenic potential (Fig. 3A–C). Immunohistochemical analysis of Ki67 expression revealed that DCAF4 silencing suppressed HCC cell proliferation. TUNEL staining indicated an increase in apoptotic cells upon DCAF4 knockdown. Importantly, NAC treatment effectively mitigated apoptosis and concomitantly increased Ki67 expression in tumors derived from DCAF4-knockdown cells (Fig. 3D). DHE staining of tumor sections across different experimental groups demonstrated that both sh-1 and sh-2 exhibited significantly higher levels of ROS compared to the sh-NC group. Notably, administration of NAC effectively abrogated the DCAF4 knockdown-induced elevation of intratumoral ROS (Fig. 3E and F). Conversely, we observed the opposite effects in tumors overexpressing DCAF4 using an established in vivo model of BT-induced oxidative stress (Fig. S3A–E). In the orthotopic model, DCAF4 overexpression enhanced intrahepatic tumor growth, whereas its knockdown suppressed it. NAC treatment rescues the suppression of tumor growth following DCAF4 knockdown (Fig. 3G and H). To further validate the impact of DCAF4 on HCC tumorigenesis, we employed a chemical-induced hepatocarcinogenesis model, administering AAV8-TBG-cre or AAV8-TBG-ctrl viruses to the mice (Figs. 3I and S3F). H&E staining and microMRI revealed the hallmark pathological and imaging features of HCC, confirming the efficacy of the chemical carcinogenesis protocol (Fig. 3J). The knockdown efficiency of AAV8-TBG-cre in liver tissues was confirmed by immunoblotting (Fig. S3G). Consistent with these findings, in DEN–CCl4-induced HCC mice, DCAF4 knockdown resulted in a significant reduction in both tumor number and size (Fig. 3K and L).

### E3 ligase complex CUL4/DDB1/DCAF4 Interacts with KEAP1 to regulate ROS

**Fig. 4:**
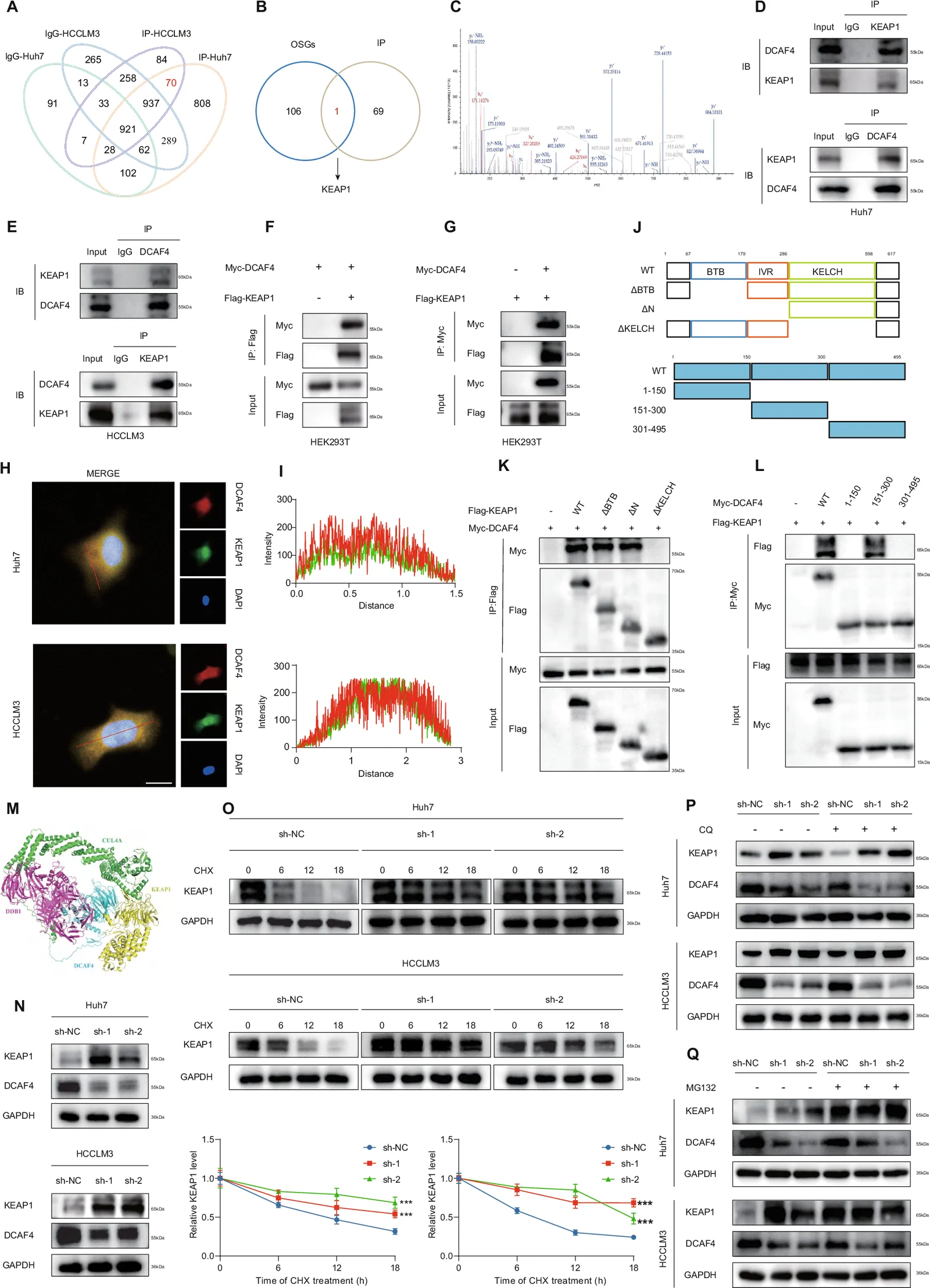
DCAF4 interacts with and promotes KEAP1 degradation. **A** Identification of DCAF4-interacting proteins by mass spectrometry (MS). **B** Venn diagram depicting the overlap between oxidative stress-related genes (OSGs) and the DCAF4 interactome. **C** MS analysis confirming KEAP1 presence in DCAF4 immunoprecipitates. **D** and **E** Co-immunoprecipitation (Co-IP) assays validating the DCAF4-KEAP1 interaction in Huh7 and HCCLM3 cells. **F** and **G** Co-IP analysis of the DCAF4-KEAP1 interaction in HEK293T cells transfected with Myc-DCAF4 and Flag-KEAP1. **H** and **I** Immunofluorescence images showing DCAF4 and KEAP1 co-localization in Huh7 and HCCLM3 cells. Scale bars: 20 μm. **J** Schematic of DCAF4 and KEAP1 truncation mutants. **K** Co-IP and immunoblotting (IB) analysis of HEK293T cells expressing DCAF4 and Flag-tagged KEAP1 truncations. **L** Co-IP and IB analysis of HEK293T cells expressing KEAP1 and Myc-tagged DCAF4 truncations. **M** Proposed model of the CUL4A/DDB1/DCAF4 E3 ligase complex-KEAP1 binding mode. **N** KEAP1 protein levels in control versus DCAF4-knockdown Huh7 and HCCLM3 cells. **O** KEAP1 protein levels upon DCAF4 knockdown in Huh7 and HCCLM3 cells treated with cycloheximide (CHX, 100 μg/mL) for the indicated durations. **P** and **Q** IB analysis of KEAP1, DCAF4, and GAPDH in Huh7 and HCCLM3 cells transduced with sh-NC, sh-1, or sh-2 and treated with MG132 (10 μM) or chloroquine (CQ, 50 μM). *P < 0.05; **P < 0.01; ***P < 0.001.

To elucidate the mechanistic basis through which DCAF4 modulates redox homeostasis, Co-IP assays were performed in HCCLM3 and Huh7 cells using a DCAF4-specific antibody. Comparative proteomic profiling of the immunoprecipitated complexes via mass spectrometry revealed 70 candidate interacting proteins common to both cell lines, as depicted in the Venn diagram (Fig. 4A). Concurrently, 107 oxidative stress-related genes (OSGs) were retrieved from the GeneCards database (https://www.genecards.org/), applying a relevance score threshold of ≥25. To delineate the specific mechanistic target through which DCAF4 modulates oxidative stress, we cross-referenced the list of OSGs with the putative DCAF4-interacting proteins. This integrated analysis identified KEAP1 as a high-priority candidate for further experimental validation (Fig. 4B and C). KEAP1 serves as a central regulator of the NRF2 pathway, which is critically involved in the cellular response to oxidative stress [17].

Co-IP assays further confirmed that both exogenous and endogenous DCAF4 interact with KEAP1 in HCC and HEK293T cells (Fig. 4D–G). Furthermore, this interaction was supported by immunofluorescence staining demonstrating cytoplasmic co-localization of DCAF4 and KEAP1 (Fig. 4H and I). To delineate the functional domains responsible for the DCAF4–KEAP1 interaction, we generated a series of truncated mutants of both proteins (Fig. 4J). Exogenous Co-IP analyses indicated that the KELCH domain of KEAP1 is critical for binding to the 151-300 amino acid region of DCAF4 (Fig. 4K and L). Previous studies have reported that DCAF4 interacts with DDB1 and CUL4A to form an E3 ubiquitin ligase complex [10, 18]. Molecular docking models based on AlphaFold3 revealed that KEAP1 binds to the CUL4/DDB1/DCAF4 complex. This binding is stabilized by multiple hydrogen bonds and hydrophobic interactions at the interface (Fig. 4M). Given that DCAF4 has been implicated as a substrate receptor for the E3 ubiquitin ligase complex that promotes target protein degradation, we investigated whether DCAF4 influences KEAP1 expression. Knockdown of DCAF4 markedly increased KEAP1 protein levels (Fig. 4N). Consistent with this, cycloheximide (CHX) chase assays showed that depletion of DCAF4 attenuated the degradation of endogenous KEAP1, indicating that DCAF4 shortens the half-life of KEAP1 (Fig. 4O). To explore whether DCAF4 facilitates KEAP1 degradation via the ubiquitin-proteasome pathway or the autophagy-lysosome pathway, HCC cells were treated with the proteasome inhibitor MG132 or the lysosome inhibitor chloroquine (CQ). The increase in KEAP1 protein resulting from DCAF4 knockdown was reversed by MG132 but not by CQ, confirming that DCAF4 mediates KEAP1 degradation specifically through the ubiquitin–proteasome pathway (Fig. 4P and Q).

### Ubiquitination of KEAP1 by DCAF4 is modulated by the acetylation level of KEAP1

**Fig. 5:**
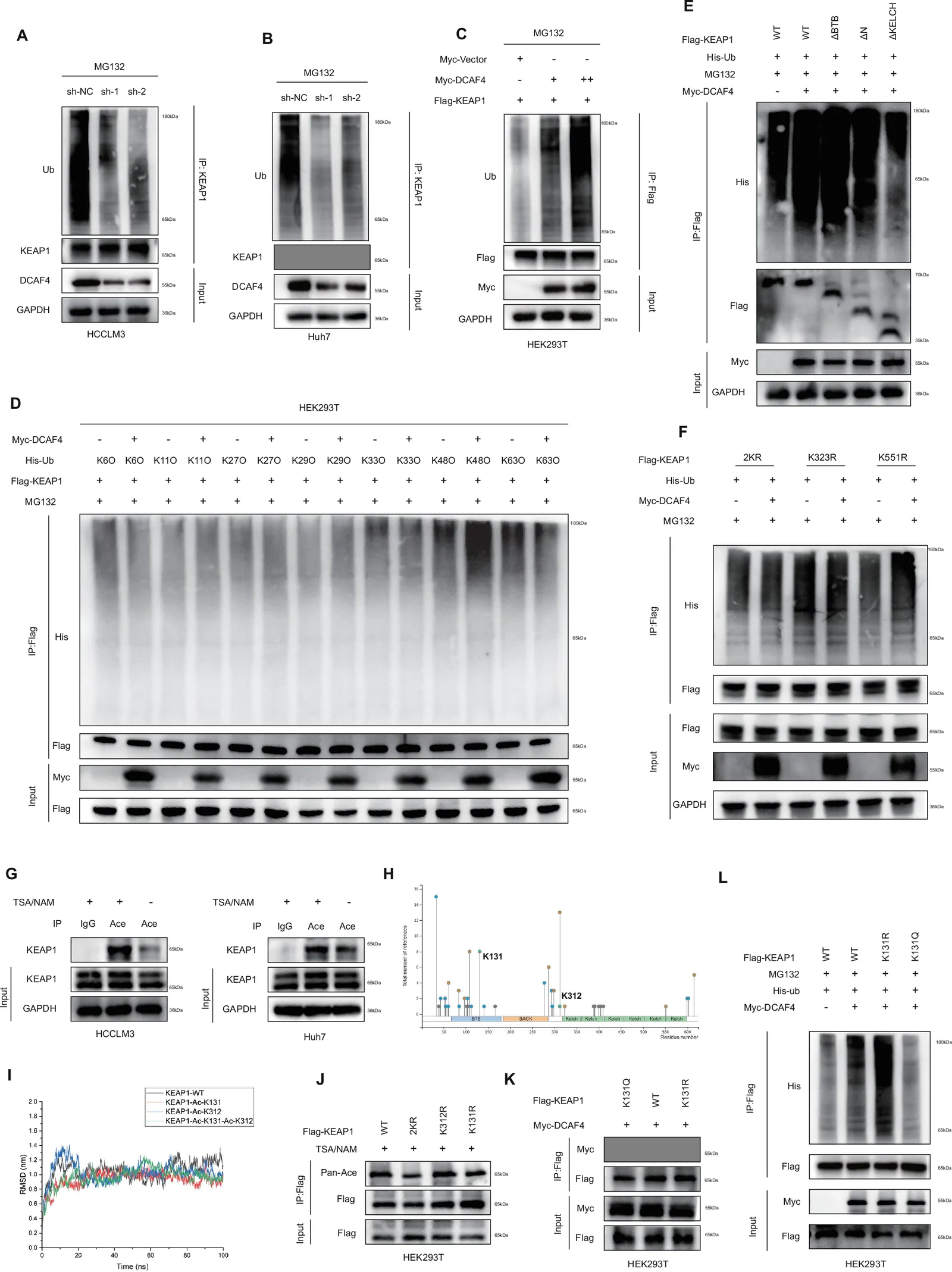
KEAP1 ubiquitination by DCAF4 is modulated by its own acetylation level. **A** and **B** Huh7 and HCCLM3 cells were transfected with sh-NC, sh-1, or sh-2 as indicated. Cell lysates were IP with an anti-KEAP1 antibody, followed by IB with anti-Ub and anti-KEAP1 antibodies. Before harvesting, cells were treated with 20 μM MG132 for 8 h. **C** HEK293T cells were co-transfected with increasing amounts of Myc-DCAF4 and a fixed amount of Flag-KEAP1. Lysates were subjected to IP using Flag beads and IB with anti-Flag and anti-Ub antibodies. Cells were treated with 20 μM MG132 for 8 h prior to collection. **D** HEK293T cells were transfected with Flag-KEAP1 and His-Ub (K6O, K11O, K27O, K29O, K33O, K48O, or K63O), along with empty vector or Myc-DCAF4 as indicated. After 48 h, cells were treated with 20 μM MG132 for 8 h. Flag-IP was performed, followed by IB with anti-His and anti-Flag antibodies. **E** Ubiquitination assays of Flag-KEAP1 and its deletion mutants in HEK293T cells co-transfected with His-Ub and Myc-DCAF4. Cells were treated with 20 μM MG132 for 8 h before analysis. **F** Ubiquitination of KEAP1 in HEK293T cells co-expressing His-Ub, Myc-DCAF4, and either Flag-KEAP1-2KR, Flag-KEAP1-K323R, or Flag-KEAP1-K551R. Cells were treated with 20 μM MG132 for 8 h. **G** Huh7 and HCCLM3 cells were treated with NAM (10 mM, 4 h) and TSA (0.2 μM, 4 h) to enhance protein acetylation. Lysates were immunoprecipitated with anti-pan-acetyllysine (Ace) antibody and immunoblotted for KEAP1. **H** Proteomic database analysis reveals two lysine residues in KEAP1 as potential acetylation sites. **I** Root-mean-square deviation (RMSD) of the protein backbone over time for non-acetylated, K131-acetylated, K312-acetylated, and K131/K312-diacetylated KEAP1. ns, nanosecond. **J** HEK293T cells expressing Flag-KEAP1 WT, 2KR, K131R, or K312R were treated with NAM (10 mM) and TSA (0.2 μM) for 4 h to promote acetylation. Flag immunoprecipitates were analyzed by IB with anti-pan-Ace and anti-Flag antibodies. **K** HEK293T cells were co-transfected with Myc-DCAF4 and either Flag-KEAP1-WT, K131R, or K131Q. Lysates were subjected to Flag-IP and immunoblotted with anti-Myc and anti-Flag antibodies. **L** Ubiquitination of KEAP1 WT, K131R, and K131Q in HEK293T cells co-expressing His-Ub and Myc-DCAF4. Cells were treated with 20 μM MG132 for 8 h before analysis.

To investigate the potential role of DCAF4 in mediating KEAP1 ubiquitination, we ectopically expressed DCAF4 in HCC cells. Immunoprecipitation of DCAF4 under MG132 treatment revealed pronounced ubiquitination of KEAP1, which was significantly diminished upon DCAF4 knockdown using two independent shRNAs (Fig. 5A and B). Furthermore, DCAF4 expression enhanced KEAP1 ubiquitination in a dose-dependent manner (Fig. 5C). CUL4A knockdown abrogated the DCAF4-mediated enhancement of KEAP1 ubiquitination (Fig. S4A), prevented the subsequent decline in KEAP1 protein (Fig. S4B), and blunted the transcriptional elevation of HO1 and NQO1 caused by DCAF4 overexpression (Fig. S4C). To determine the specific linkage type implicated in DCAF4-mediated KEAP1 regulation, we utilized a panel of ubiquitin mutants in which all lysines except one were substituted with arginine. As shown in Fig. 5D, DCAF4 specifically promoted K48-linked polyubiquitination of KEAP1. To map the ubiquitination sites on KEAP1, we generated a series of truncation mutants based on its domain structure. These assays indicated that regions containing the KELCH domain were subject to DCAF4-dependent ubiquitination (Fig. 5E). We then systematically mutated each lysine within this domain to arginine, preserving one lysine at a time for functional assessment. Based on proteomic database screening (https://www.phosphosite.org/), two potential ubiquitination sites, Lys323 and Lys551, were identified and found to be evolutionarily conserved (Fig. S4D and E). Site-directed mutagenesis followed by ubiquitination assays revealed that Lys323 serves as the primary residue for DCAF4-mediated ubiquitination of KEAP1 (Fig. 5F).

Previous studies have suggested that acetylation of oncoproteins often enhances their stability and promotes oncogenic functions[19]. We observed that treatment with trichostatin A (TSA) and nicotinamide (NAM) increased the acetylation level of KEAP1 (Fig. 5G). Proteomic database analysis further identified two conserved acetylation sites, Lys131 and Lys312 (Figs. 5H and S4F). The molecular dynamics simulations revealed that acetylation at K131 reduces RMSD fluctuations, suggesting enhanced structural stability compared to the wild-type (Fig. 5I). Using single (K131R, K312R) and double (K131/312R) acetylation-defective mutants, we demonstrated that acetylation occurs predominantly at Lys131 (Fig. 5J). Acetylation can alter a protein’s three-dimensional structure, thereby influencing its interactions with other proteins [20]. We further considered whether increased levels of KEAP1 acetylation would affect DCAF4 binding and the consequent elevation in its mediated ubiquitination levels. Next, we ectopically expressed KEAP1 WT, a simulated acetylated K131Q mutant, or an acetylation-deficient K131R mutant in HEK293T cells. Compared with KEAP1 WT, ectopic expression of KEAP1 131Q inhibited the binding of KEAP1 to DCAF4 and effectively reversed the increase in DCAF4-mediated ubiquitination levels (Fig. 5K and L).

### Stress granules provide a platform for DCAF4 binding to KEAP1 under oxidative stress

KEAP1 serves as a key negative regulator of NRF2, a central transcription factor in the cellular antioxidant response pathway [21]. To investigate whether DCAF4 functions through the NRF2 pathway, we treated HCC cells with an NRF2 inhibitor, ML385, and observed that it significantly attenuated the oxidative stress resistance conferred by DCAF4 overexpression, while also promoting intracellular ROS accumulation (Fig. S5A and B). Further colony formation confirmed that NRF2 inhibition markedly impaired the protective effect of DCAF4 on cell survival, indicating that DCAF4-mediated antioxidant activity is highly dependent on the NRF2 pathway (Fig. S5C and D).

**Fig. 6:**
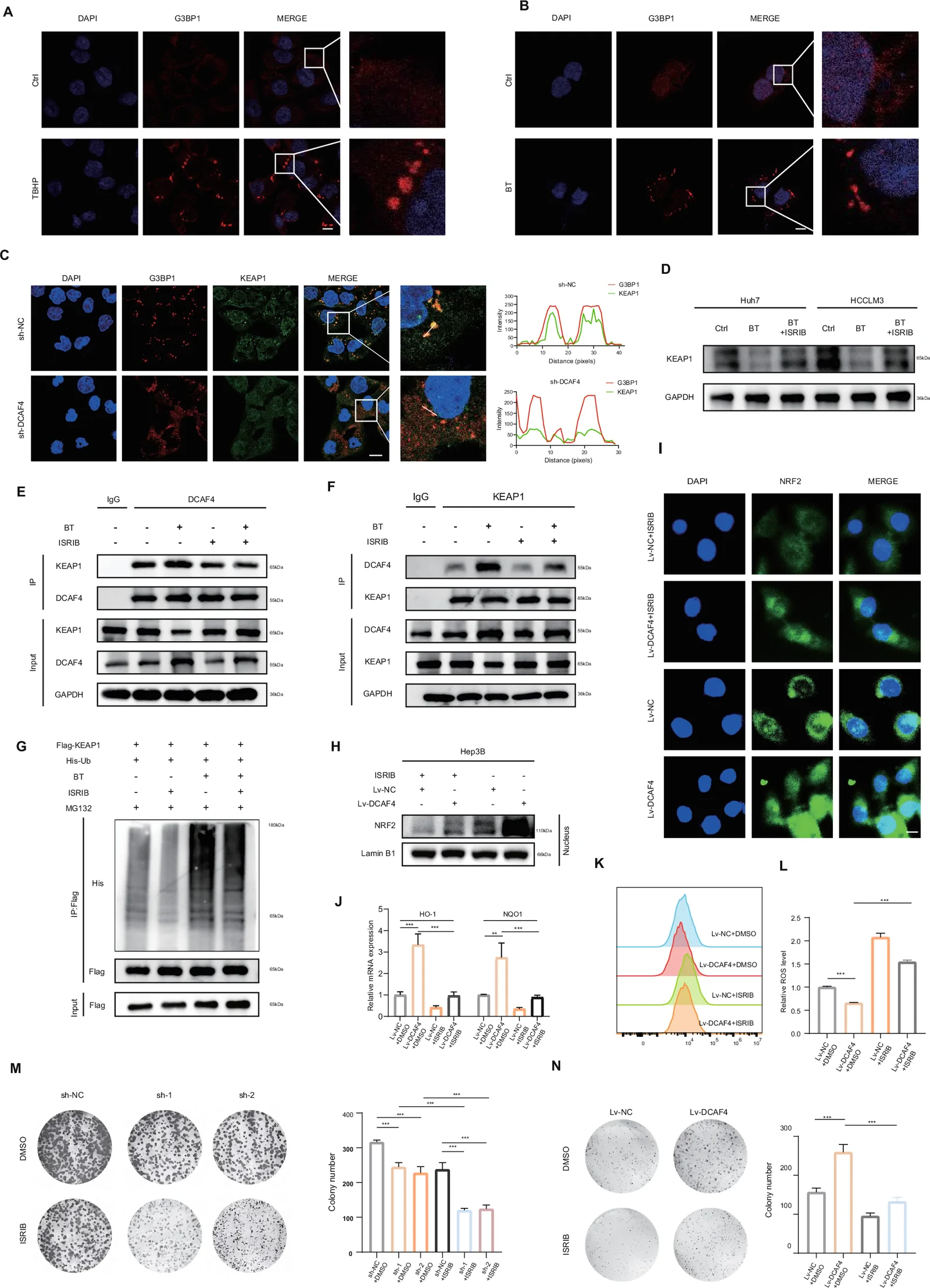
Stress granule assembly is critical for DCAF4 in the activation of NRF2. **A** and **B** Immunofluorescence images depicting G3BP1 and KEAP1 localization in Huh7 and HCCLM3 cells following TBHP (10 μM, 1 h) or BT. Scale bars: 10 μm. **C** Immunofluorescence analysis of G3BP1 and KEAP1 in Huh7 cells transfected with sh-NC or sh-DCAF4 and treated with BT. Line-scan profiles reflect fluorescence intensities along the indicated path. Scale bars: 10 μm. **D** Western blot analysis of KEAP1 in HCC cells treated with BT or BT + ISRIB(200 nM). **E** and **F** Co-IP analysis of the DCAF4-KEAP1 interaction in HCC cells. **G** Ubiquitination of KEAP1 in HEK293T cells co-expressing His-Ub and Flag-KEAP1. **H** Nuclear fractionation and western blot analysis of NRF2 in control and DCAF4-overexpressing Hep3B cells treated with ISRIB (200 nM) under BT. LaminB1 was used as a nuclear marker. **I** Subcellular localization of NRF2 in control and DCAF4-overexpressing Hep3B cells following ISRIB treatment (200 nM) under BT. Nuclei were stained with DAPI. Scale bar: 10 μm. **J** Relative mRNA levels of HO1 and NQO1 in control and DCAF4-overexpressing Hep3B cells, treated with or without ISRIB (200 nM) under BT (*n* = 3). **K** and **L** Flow cytometry (left) and quantification (right) of intracellular ROS levels assessed by DCFH-DA staining in DCAF4-overexpressing Hep3B cells treated with ISRIB (200 nM) under BT (*n* = 3). **M** and **N** Representative images (left) and quantitative analysis (right) of colony formation assays in DCAF4-silenced Huh7 cells and DCAF4-overexpressing Hep3B cells treated with ISRIB (200 nM) under BT (*n* = 3). *P < 0.05; **P < 0.01; ***P < 0.001.

SGs are dynamic, membraneless organelles that assemble through liquid–liquid phase separation (LLPS) and have been implicated in various cellular stress responses, including cancer drug resistance [22]. Notably, LLPS of KEAP1 generates condensates that sequester KEAP1, thereby compartmentalizing it to facilitate NRF2 activation [23, 24]. Inspired by this parallel, we sought to investigate whether SGs play a comparable role in regulating the NRF2 pathway under oxidative stress. Using confocal microscopy, we first examined the dynamics of the canonical SG marker G3BP1 and confirmed that TBHP or BT-induced oxidative stress robustly promoted SG assembly (Fig. 6A and B). Importantly, we observed recruitment of KEAP1 and DCAF4 into these stress-induced structures. Subsequent immunofluorescence analysis revealed that knockdown of DCAF4 markedly reduced the co-localization of KEAP1 with G3BP1-positive SGs, indicating that DCAF4 is critically required for the efficient recruitment of KEAP1 to SGs (Figs. 6C and S5E–G). BT induces KEAP1 degradation, which can be reversed by SGs inhibitor ISRIB or G3BP1 knockdown (Figs. 6D and S5H). Further investigation revealed that the interaction between DCAF4 and KEAP1 is enhanced under oxidative stress in a manner dependent on SGs formation (Figs. 6E, F and S5I, J). Inhibition of SGs assembly abolishes the upregulation of KEAP1 ubiquitination mediated by DCAF4 (Fig. 6G). These findings indicate that the promotion of KEAP1 ubiquitination and degradation by DCAF4 under stress conditions relies on the formation of SGs.

We next examined whether DCAF4 and SG assembly jointly regulate NRF2 nuclear translocation and transcriptional activity. Western blot showed that inhibition of SGs diminishes the DCAF4 overexpression or BT-induced nuclear accumulation of NRF2 (Figs. 6H and S5K, L). Immunofluorescence demonstrated the accumulation of nuclear NRF2 in different treatment groups (Fig. 6I). qRT-PCR results demonstrated that inhibition of SG reversed the increased transcriptional levels of the NRF2 direct target genes HO-1 and NQO1 induced by DCAF4 overexpression, under oxidative stress (Fig. 6J). ISRIB treatment attenuated the protective effect of DCAF4 overexpression against oxidative stress and enhanced the accumulation of ROS induced by DCAF4 knockdown (Figs. 6K, L and S5M, N). Knockdown of G3BP1 potentiates BT-induced increases in ROS levels and apoptosis (Fig. S5O–R). The pro-survival function of DCAF4, as demonstrated by colony formation, was markedly impaired upon SG inhibition (Fig. 6M and N), thereby establishing SG formation as an integral component of the DCAF4-driven antioxidant defense mechanism.

### XBP1 activates DCAF4 transcription in response to ROS signaling

Using TF-Target Finder, we performed transcription factor prediction for DCAF4 across four relevant datasets. The overlapping predictions included XBP1, KLF5, MYC, and MYCN (Fig. S6A). XBP1 activation upon oxidative stress is a key cytoprotective mechanism that drives cellular adaptation. As a master transcriptional regulator, XBP1 orchestrates downstream stress-responsive gene expression [25]. Given the potential role of DCAF4 in mediating oxidative stress responses in HCC, we hypothesized that its expression is functionally linked to the activation of XBP1 under such conditions. To test this, we induced oxidative stress in HCC cell lines using TBHP and quantified DCAF4 expression at multiple dose points. Both qRT-PCR and Western blot analyses indicated that DCAF4 expression was upregulated in a time-dependent manner (Fig. S6B–E). The regulatory role of XBP1 in modulating DCAF4 expression under oxidative stress was examined via qRT-PCR and Western blotting. Results indicated that knockdown of XBP1 led to a marked reduction in DCAF4 levels in HCC cells (Fig. S6F–I). CUT&Tag sequencing results showed that an obvious binding peak of XBP1 was observed in the DCAF4 promoter region (Fig. S6J). Using the JASPAR online tool, we identified three putative XBP1 binding sites in the genomic region adjacent to the DCAF4 transcription start site (NC_000014) (Fig. S6K). Sequential mutagenesis of each site and subsequent luciferase reporter assays revealed that mutation of Binding site 1 abrogated the modulation of luciferase activity by XBP1 overexpression, an effect not observed with mutations at the other two sites (Fig. S6L). chromatin immunoprecipitation (ChIP) assays confirmed significant enrichment of chromatin fragments containing binding site 1 in XBP1 overexpression (Fig. S6M). To understand the correlation between XBP1 and DCAF4 in HCC tissues, the same tissue array was used to detect the expression levels of XBP1 and DCAF4 protein by immunofluorescence. It was observed that the levels of DCAF4 were relatively higher with XBP1 (Fig. S6N and O). In addition, HCC data mining of the TCGA database demonstrated a positive correlation between expression of XBP1 and DCAF4 (Fig. S6P). Our data define XBP1 as the critical transcription factor responsible for stress-induced DCAF4 expression in HCC, thereby elucidating the significance of this regulatory axis in the disease pathogenesis.

### Discovery of a small-molecule inhibitor targeting the active site of DCAF4

**Fig. 7:**
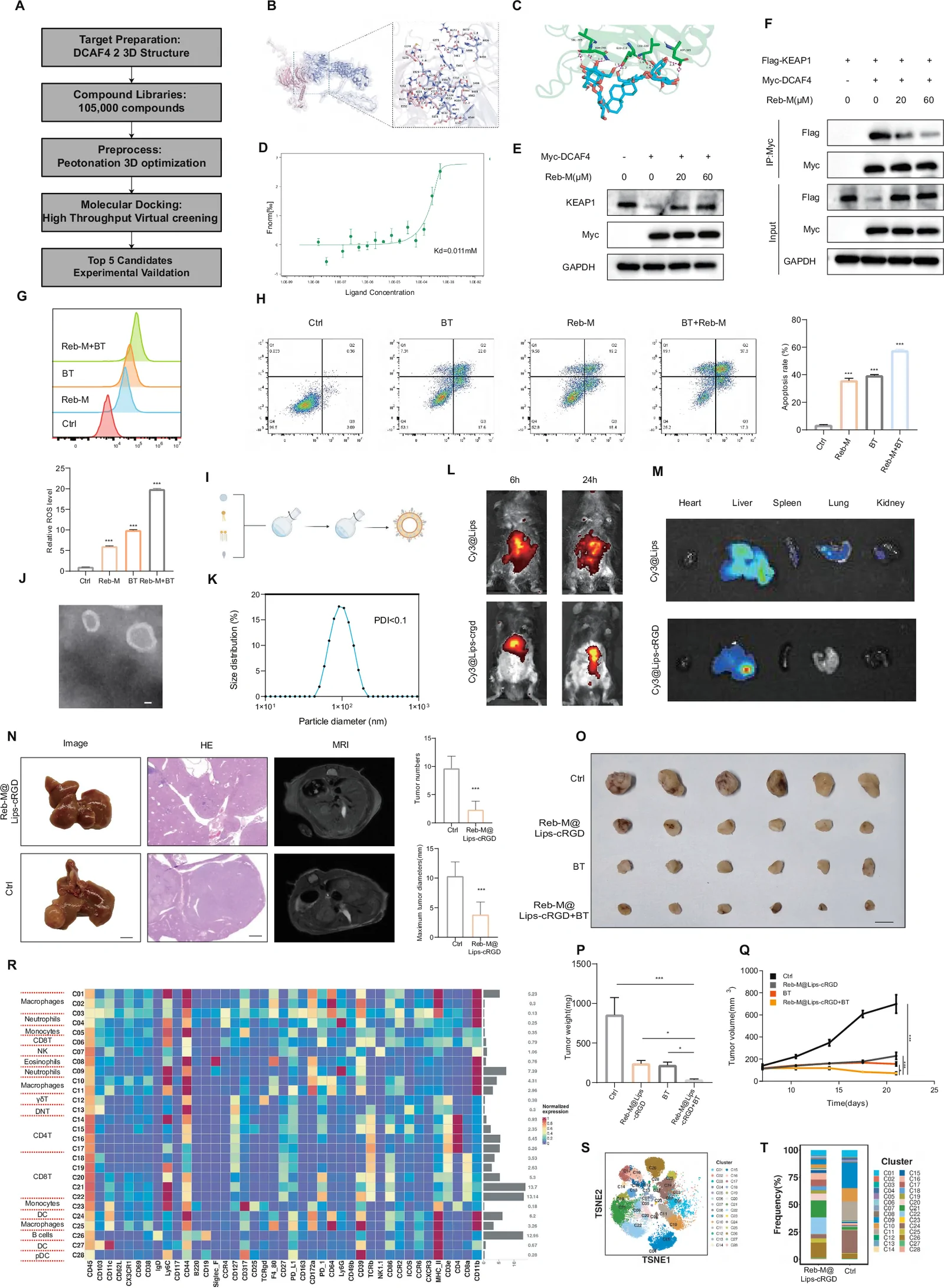
Identification of a small molecular inhibitor targeting DCAF4. **A** Flowchart of virtual screening for small-molecule inhibitors targeting DCAF4–KEAP1 interaction. **B** Postulated modes of binding between DCAF4 and KEAP1. **C** A simplified depiction of Reb-M occupying the DCAF4 protein’s active site. **D** MST analysis quantified the binding affinity between Reb-M and DCAF4. **E** Western blot analysis of KEAP1 protein levels in HEK293T cells after 48 h of treatment with varying concentrations of Reb-M. **F** Co-IP was conducted after 48 h of treatment with different concentrations of Reb-M following the transfection of FLAG-KEAP1 and Myc-DCAF4 into HEK293T cells. **G** Flow cytometric analysis and quantification of Huh7 cells treated with BT, Reb-M, or BT+Reb-M, showing intracellular ROS levels via DCFH-DA staining (*n* = 3). **H** Apoptosis in Huh7 cells treated with Ctrl, BT, Reb-M, or BT+Reb-M, analyzed by flow cytometry using an Annexin V/PI Apoptosis Detection Kit (*n* = 3). **I** Reb-M@Lips-cRGD preparation workflow diagram. **J** Representative transmission electron microscopy (TEM) images displaying the morphology of the modified lipid nanoparticles. Scale bars: 20 nm. **K** Dynamic light scattering (DLS) analysis demonstrating the size distribution of the nanoparticles. **L** and **M** Fluorescence imaging of in vivo and ex vivo distributions of nanoparticles in organs. **N** Representative macroscopic images (scale bars: 5 mm), HE staining (scale bars: 1 mm), and microMRI of livers from Ctrl and Reb-M@Lips-cRGD treated mice. Tumor volumes and weights are shown on the right. **O**–**Q** C57BL/6J mice treated with Ctrl, BT, Reb-M@Lips-cRGD, or BT+Reb-M@Lips-cRGD, followed by a tumor formation assay (scale bars: 10 mm). Tumor growth and weight were statistically analyzed in the subcutaneous Hepa1-6 xenograft model. **R** 28 cell clusters in total were defined in the respective groups. **S** TSNE plot showing distributions of 28-cell clusters. **T** The plot visualized the proportions of cell clusters in Ctrl and Reb-M@Lips-cRGD group. *P < 0.05; **P < 0.01; ***P < 0.001.

Given the crucial oncogenic role of the DCAF4/KEAP1 signaling axis in HCC, which is mediated through the regulation of redox homeostasis, the discovery of inhibitors targeting the DCAF4–KEAP1 protein–protein interaction interface has garnered significant attention. We hypothesized that key druggable sites might exist within the DCAF4 region responsible for KEAP1 binding. Using Schrödinger Maestro 12.8 software, we computationally predicted the potential active pockets at this interaction interface. Our structural analysis revealed a promising binding pocket on the DCAF4 protein surface, characterized by critical residues such as GLU218 and LEU220. Subsequently, we performed molecular docking screens of over 105,000 compounds against this predicted pocket. Based on the top-ranking binding poses and scores from the DCAF4-compound interaction models (Fig. 7A and B), we selected and acquired the five highest-ranked compounds for further experimental validation. Among these, Rebaudioside M (Reb-M) demonstrated a favorable binding mode, forming multiple hydrogen bonds with key amino acid residues within the DCAF4 interaction domain, including VAL265, ASN266, GLU218, LEU220, and ASP165. Specific bond distances (Å) are detailed in the corresponding 3D interaction diagram (Figs. 7C and S7A). Microscale thermophoresis (MST) is a powerful technique for analyzing biomolecular interactions and has proven particularly effective in validating the binding of small-molecule inhibitors to their target proteins, offering distinct advantages in addressing specific challenges in drug discovery [26]. MST results showed that Reb-M binds to DCAF4, with a dissociation constant (*K*_d_) corresponding to a binding affinity of ~11 µM (Fig. 7D). To further elucidate the functional role of Reb-M, we investigated its effect on the DCAF4–KEAP1 interaction in HCC cells. We found that treatment with Reb-M rescued the DCAF4-induced downregulation of KEAP1 protein levels in HCC cells (Fig. 7E). Co-IP assays further demonstrated that Reb-M treatment resulted in a dose-dependent reduction in the binding between Flag-tagged KEAP1 and Myc-tagged DCAF4 (Fig. 7F).

BT, including modalities such as 90Y microsphere radioembolization, ^125^I seed implantation, and radioactive iodine-labeled lipiodol, is widely used in the comprehensive management of HCC [27, 28]. A key mechanism underlying BT-induced cancer cell death involves the substantial generation of ROS [13, 29]. To investigate whether Reb-M enhances the sensitivity of HCC to BT, we established an in vitro model using ^125^I seeds [30]. Flow cytometry analysis revealed that the combination of Reb-M and BT induced significantly higher ROS production compared to BT alone (Fig. 7G). Additionally, apoptosis assays showed that Reb-M markedly enhanced BT-induced apoptosis in HCC cells (Fig. 7H). To enhance the hepatic targeting and in vivo stability of Reb-M, we developed a tumor-cell-intrinsic Reb-M liposomal nanoparticle platform termed Reb-M@Lips-cRGD specifically designed for the targeted delivery of DCAF4 inhibitors, and evaluated its efficacy in suppressing HCC growth (Fig. 7I). The physicochemical properties of the synthesized nanoliposomes were systematically characterized (Fig. S7B and C). Transmission TEM imaging confirmed the typical nanostructure of Reb-M@Lips-cRGD (Fig. 7J), with a hydrodynamic diameter of approximately 107.2 ± 2.4 nm (Fig. 7K). In vivo and ex vivo fluorescence imaging further demonstrated significant preferential accumulation of Reb-M@Lips-cRGD in liver tumor regions compared to normal tissues such as the heart and spleen, indicating excellent tumor-targeting capability (Fig. 7L and M).

To evaluate the therapeutic efficacy, we investigated the inhibitory effect of Reb-M@Lips-cRGD on DCAF4 and its combined antitumor activity with BT in vivo. The results showed that Reb-M@Lips-cRGD not only effectively suppressed HCC progression but also significantly enhanced the efficacy of BT. The combined treatment group exhibited markedly reduced tumor weight and volume, along with significantly inhibited tumor growth rates (Fig. 7N–Q). Immunohistochemical analysis further confirmed that Reb-M@Lips-cRGD demonstrated the most pronounced effect in inhibiting HCC cell proliferation and inducing apoptosis (Fig. S7D). In terms of biosafety, we assessed the cytotoxicity of Reb-M@Lips-cRGD and confirmed that it exhibited no significant toxicity in mice at the experimental dosage. After three weeks of intravenous administration via the tail vein in healthy mice, blood biochemical analysis revealed that levels of alanine aminotransferase (ALT), aspartate aminotransferase (AST), and body weight remained within normal physiological ranges (Fig. S7E–G). Moreover, histopathological examination of the heart, liver, spleen, lungs, and kidneys showed no obvious pathological changes (Fig. S7H). In HCC, dysregulated redox states directly shape the immune landscape within the tumor microenvironment (TME) [31]. To further explore the influence of Reb-M@Lips-cRGD on the HCC immune microenvironment, tumor samples from Hepa1-6 subcutaneous tumor-bearing C57BL/6J mice were subjected to high-dimensional single-cell mass cytometry analysis, comparing control and Reb-M@Lips-cRGD-treated groups. From dissociated tumor tissues, we isolated single, viable, and intact CD45^+^ immune cells for analysis. Unsupervised clustering identified 28 distinct immune subsets, which were annotated based on canonical marker expression (Figs. 7R and S8A). Comparative assessment revealed that Reb-M@Lips-cRGD substantially remodeled immune cell composition: the proportions of immunosuppressive populations, including M2 macrophages, neutrophils, monocytes, and MHCII^−^ macrophages, were significantly reduced. Conversely, we observed a marked increase in plasmacytoid dendritic cells (pDCs), B cells, eosinophils, and both CD8^+^ and CD4⁺T cells (Figs. 7S and S8B). At the molecular level, immune-related markers such as CD86 and ICOS were upregulated in Reb-M@Lips-cRGD group, whereas PD-L1 expression was downregulated (Fig. S8C–F). In summary, the Reb-M@Lips-cRGD delivery system developed in this study demonstrates favorable targeting capability, therapeutic efficacy, and biosafety when combined with ^125^I-BT for HCC, showing promising potential for clinical application.

## Discussion

HCC remains a highly lethal malignancy worldwide, with high recurrence rates and limited therapeutic efficacy [32]. A key challenge in its clinical management, particularly for locoregional modalities such as BT, is the frequent development of radioresistance [33]. BT exerts its antitumor effects largely through the generation of ROS, which induce lethal DNA damage and trigger apoptotic cell death [4]. Thus, oxidative stress represents a dual-faced player in HCC therapy. Although radiotherapy intentionally elevates ROS to eradicate malignant cells, HCC tumors often activate adaptive antioxidant responses that counteract this cytotoxic insult [34, 35]. A central mechanism of radioresistance involves the upregulation of intrinsic antioxidant systems, which scavenge therapy-induced ROS and sustain tumor survival and progression [36]. Therefore, targeting these redox adaptive pathways offers a promising strategy for restoring radiosensitivity in HCC. The role of DCAF4, known as a component of the CRL4 ubiquitin ligase complex, in the pathogenesis of HCC has yet to be thoroughly explored [8]. In this study, we demonstrate that DCAF4 is highly expressed in HCC and promotes NRF2 activation through ubiquitinating KEAP1 and modulating its sequestration into SGs. This signaling cascade leads to transcriptional upregulation of key antioxidant genes, including HO-1 and NQO-1, thereby attenuating stress-induced ROS accumulation and subsequent cell death. Through structure-based virtual screening, we identified Reb-M as a small-molecule inhibitor that specifically targets the DCAF4-KEAP1 binding interface. This compound demonstrated efficacy in both in vitro and in vivo settings by modulating ROS homeostasis and consequently conferring enhanced radiosensitivity.

The KEAP1/NRF2 signaling pathway orchestrates cellular defense mechanisms against oxidative and electrophilic stress [37]. Upon exposure to exogenous or intrinsic oxidative insults, cells upregulate NRF2 expression through facilitated degradation of KEAP1 protein. The activated NRF2 subsequently translocates into the nucleus, initiating transcriptional programs of multiple antioxidant genes, including HO-1 and NQO1, thereby eliminating excessive ROS [38]. This pathway occupies a central position in maintaining cellular redox homeostasis, establishing it as a critical regulatory node in the pathogenesis of various diseases. Accumulating evidence has confirmed the involvement of the KEAP1/NRF2 system in resistance mechanisms across multiple cancer types, including radiation therapy resistance [39, 40]. Consistent with our findings, suppression of DCAF4 expression elevates the oxidative burden, thereby enhancing the sensitivity of HCC cells to BT. The cellular response to oxidative stress involves an intricate network of signaling pathways. While our study focuses on the DCAF4/KEAP1/NRF2 axis, our transcriptomic data indicate that DCAF4 is associated with the p53 signaling pathway. p53 is a well-known redox-sensitive transcription factor that can be activated by elevated ROS, leading to cell cycle arrest or apoptosis [41]. Conversely, high DCAF4 expression in HCC may keep ROS low and thereby suppress p53-dependent tumor surveillance. Although we did not directly measure p53 activity or its target genes in this study, the RNA-seq data provide a strong rationale for future investigations into whether DCAF4 modulates p53 via ROS-dependent or ROS-independent mechanisms. Another key player in redox regulation is p62 (SQSTM1), an autophagy receptor that directly binds to KEAP1 and competes with NRF2, leading to NRF2 stabilization [21]. DCAF4 acts as a substrate receptor for the CRL4 E3 ligase complex, promoting K48-linked ubiquitination and proteasomal degradation of KEAP1. This raises the possibility of functional interplay or redundancy between the p62-mediated competitive inhibition and DCAF4-mediated ubiquitination of KEAP1. Collectively, these considerations place the DCAF4/KEAP1/NRF2 axis within a broader signaling landscape, where it may work in concert with or parallel to p53 and p62 to shape the redox-adaptive capabilities of HCC cells.

Ubiquitination represents an evolutionarily conserved post-translational regulatory mechanism whose dynamic processes are precisely controlled and play pivotal roles in numerous pathological conditions. All seven lysine residues within ubiquitin molecules can be modified to form distinct types of polyubiquitin chains, ultimately determining the fate of substrate proteins [42]. Crosstalk between post-translational modifications constitutes a fundamental mechanism regulating protein stability and functionality. Our research systematically elucidates that DCAF4-mediated ubiquitination of KEAP1 primarily occurs at Lys323, while KEAP1 acetylation is localized to Lys131. Acetylation and ubiquitination primarily regulate cellular functions by modulating protein stability. Although both modifications typically target lysine residues and frequently compete for the same sites [43], our study reveals that KEAP1 acetylation can significantly influence its interaction with DCAF4 and subsequent ubiquitination, even without direct competition for identical lysine residues. This finding suggests that acetylation may function as a molecular switch that fine-tunes KEAP1 functionality by modulating its binding affinity for DCAF4. Although the detailed molecular mechanisms warrant further investigation, our study provides crucial mechanistic insights into the signal-dependent regulation of the DCAF4–KEAP1 axis.

SGs are membraneless cytoplasmic organelles consisting mainly of translationally stalled mRNAs and associated proteins. Under stress conditions such as radiotherapy, hypoxia, or heat shock, cells promote SGs formation to protect untranslated mRNAs from degradation [44]. Following stress relief, SGs rapidly disassemble, enabling mRNA release and restoration of protein synthesis [45]. As highly dynamic structures, SGs thus facilitate cell survival under adverse conditions and contribute to the development of tumor drug resistance [46]. A diverse repertoire of proteins is recruited into SGs, including translation initiation factors and regulatory proteins involved in SG assembly and function. For example, RACK1 becomes sequestered within SGs under stress, which abrogates its ability to bind MTK1 and thereby suppresses activation of the MTK1-mediated stress-induced apoptosis pathway [47]. Moreover, we observed that KEAP1, a key negative regulator of NRF2, is markedly enriched in SGs under stress conditions. Based on these findings, we further investigated whether the formation of SGs is involved in regulating DCAF4-mediated activation of the NRF2 pathway and its downstream antioxidant response. Our results demonstrate that treatment with ISRIB effectively suppresses DCAF4-dependent activation of the NRF2 pathway, indicating a regulatory role for SGs formation in this process. In summary, this study reveals the critical function of SGs in modulating the DCAF4–NRF2 signaling axis and cellular antioxidant stress responses, providing new insights into the mechanisms of chemoresistance and potential targeted therapeutic strategies.

Based on our elucidation of the DCAF4/KEAP1 axis in regulating oxidative stress in HCC, developing small-molecule modulators targeting the binding domain between DCAF4 and KEAP1 represents a promising therapeutic strategy for HCC. Given that the redox state of the TME profoundly influences antitumor immunity [48], this increase in oxidative stress disrupts the intrinsic antioxidant shield that normally protects HCC cells from oxidative damage. Consequently, the weakening of this shield not only sensitizes tumor cells to therapy but also potentially reshapes the immune landscape. Mechanistically, elevated ROS levels enhance T cell recruitment and function through multiple pathways. First, ROS acts as signaling second messengers that promote chemokine production, facilitating T cell chemotaxis [49]. ROS-induced immunogenic cell death releases damage-associated molecular patterns, facilitating T cell priming [50]. In addition, NRF2 has been reported to transcriptionally regulate PD-L1 expression [51], linking oxidative stress to immune checkpoint control. Conversely, high DCAF4 expression may contribute to an immune-cold, checkpoint-rich microenvironment that favors immune evasion. Therefore, combining DCAF4 inhibitors with immune checkpoint blockade represents a promising therapeutic strategy for HCC. With growing understanding of molecular targets and their functional mechanisms, small-molecule inhibitors have emerged as a major direction in cancer intervention due to their superior efficacy and safety profile compared to conventional therapies [52]. Among the candidates, the steviol glycoside Reb-M, a non-caloric sweetener derived from Stevia rebaudiana with known potential anticancer properties, emerged as a promising hit [53, 54]. Preliminary findings indicate that Reb-M effectively disrupts the DCAF4-KEAP1 interaction, thereby attenuating oxidative stress responses in HCC cells.

Local therapies such as BT offer minimally invasive treatment options for HCC and other malignancies, enabling precise tumor targeting while reducing procedural risks and morbidity [55]. As a commonly used radioactive source in BT, ^125^I seeds are suitable for various types of cancers but present certain limitations, such as suboptimal therapeutic efficacy and limited stimulation of antitumor immune responses [56, 57]. Therefore, it is imperative to develop potent radiosensitizers to enhance the effectiveness of ^125^I seeds. Maintaining moderate ROS levels is therefore crucial for cancer cells to avoid oxidative damage. Several strategies aimed at enhancing ROS generation have been reported to improve radiosensitivity in HCC [58, 59]. Given the pivotal role of the DCAF4/KEAP1 signaling axis in regulating ROS homeostasis in HCC cells, the combination of Reb-M with BT significantly enhanced BT-induced apoptosis in HCC cells. Liposomes have emerged as attractive nanocarriers for drug delivery due to their low immunogenicity, high stability, favorable safety profile, and cost-effectiveness [60]. However, the therapeutic performance of conventional liposomal formulations is often limited by their absence of tumor-specific targeting, leading to inadequate drug accumulation at the target site. To overcome this constraint, receptor–ligand interactions have been widely leveraged to enhance targeting precision [61]. The cRGD ligand exhibits high binding affinity for the integrin αvβ3 receptor, which is overexpressed on various cancer cells, making it an ideal tool for improving tumor-selective delivery [62]. To further advance the efficacy of Reb-M-based therapy in HCC and other malignancies, we have developed a novel active-targeting approach using cRGD-modified cationic liposomes for targeted delivery of Reb-M. This approach effectively antagonizes the DCAF4/KEAP1 protein interaction in vivo and reduces oxidative stress resistance in HCC. The Reb-M@Lips-cRGD delivery system we constructed demonstrated synergistic antitumor efficacy in combination with BT in animal models. Nevertheless, the clinical translation of this system faces several challenges, including scalable manufacturing of nanoparticles, controlled drug release kinetics, and precise in vivo targeting. While liposomes demonstrate efficient drug encapsulation, achieving spatiotemporal control over drug release remains a key determinant of therapeutic efficacy and safety. Current research focuses on enhancing liposomal stability in circulation, optimizing drug release profiles, and improving targeting specificity. Furthermore, a comprehensive assessment of long-term toxicity and immunocompatibility of these nanocarriers represents a crucial aspect of future translational studies. This combined approach provides new perspectives for overcoming clinical radioresistance in HCC.

## Conclusion

**Fig. 8:**
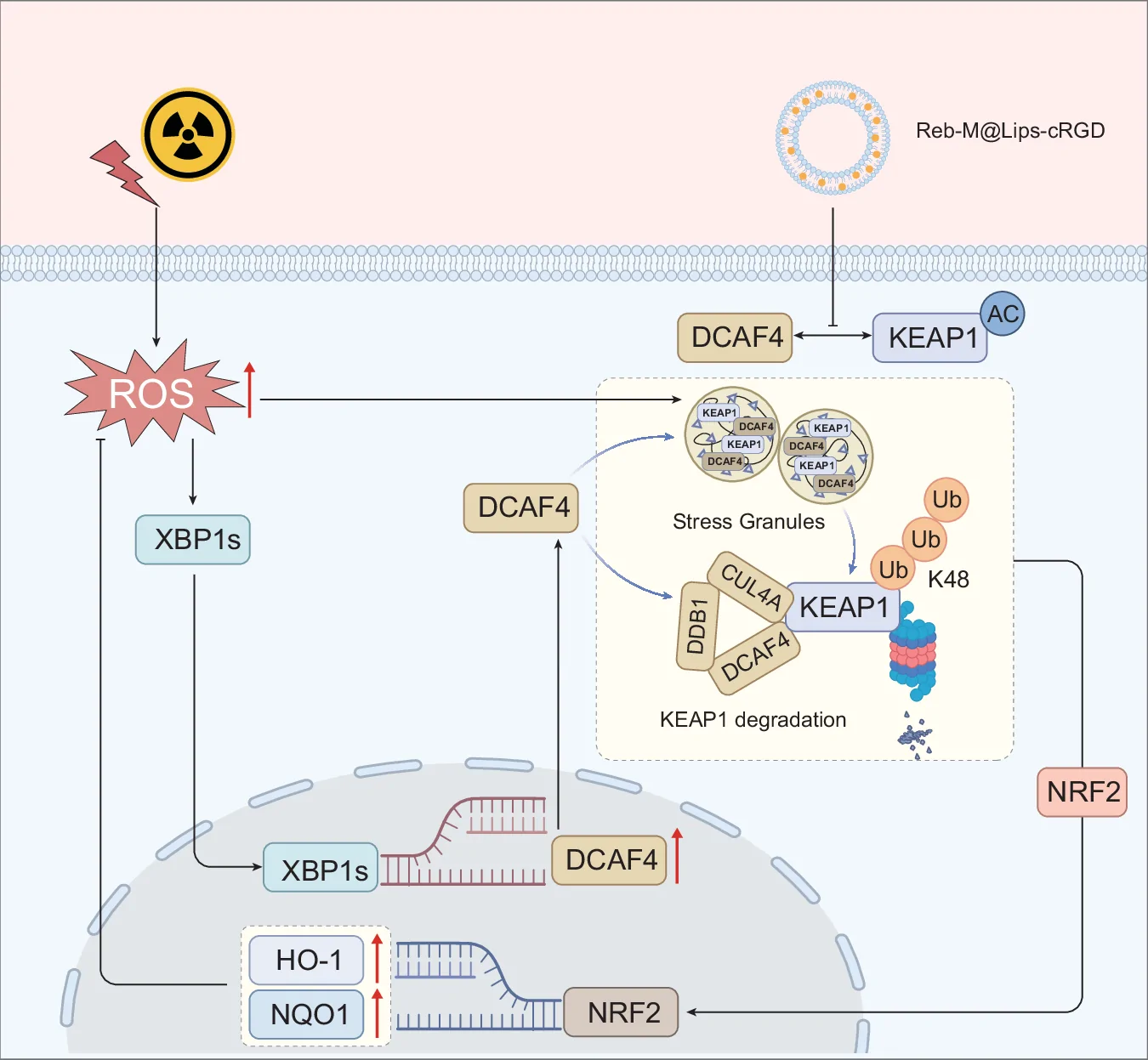
Proposed working model of the DCAF4 in regulating HCC BT-induced oxidative stress.

In summary, our study establishes the DCAF4/KEAP1/NRF2 signaling axis as a central regulatory mechanism governing redox homeostasis and radioresistance in HCC. We demonstrate that DCAF4 is induced by XBP1 under oxidative stress, drives K48-linked ubiquitination and subsequent proteasomal degradation of KEAP1, thereby facilitating NRF2-dependent transcriptional activation of antioxidant genes. This regulatory cascade is further modulated by KEAP1 acetylation and its compartmentalization into SGs. Pharmacological disruption of the DCAF4–KEAP1 interface with the small-molecule inhibitor Reb-M significantly enhances the sensitivity of HCC cells to BT, both in vitro and in vivo (Fig. 8). These findings not only identify DCAF4 as a critical mediator of adaptive oxidative stress response but also offer a novel targeted strategy to overcome radiotherapy resistance in HCC.

## Supplementary information

Supplementary methods and figure legends

Figure S1

Figure S2

Figure S3

Figure S4

Figure S5

Figure S6

Figure S7

Figure S8

WB supplementary material

## Data Availability

All data that support the findings of this study are publicly available within this article and its supplementary information. The original source data for all figures are stored by the authors and will be provided by the corresponding author upon reasonable request.
