# Supplementary material for: CRISPR/Cas9 screen identifies DCAF4 as a novel protector of hepatocellular carcinoma against brachytherapy via stress granule-dependent NRF2 activation
Source: Cell Death Dis. 2026 Jul 14;17(1):818. doi: 10.1038/s41419-026-09095-0 (PMC13594110; doi:10.1038/s41419-026-09095-0)

Figure1O

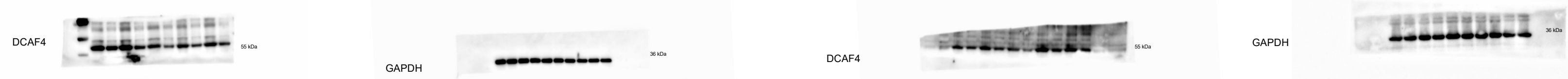

Figure4D

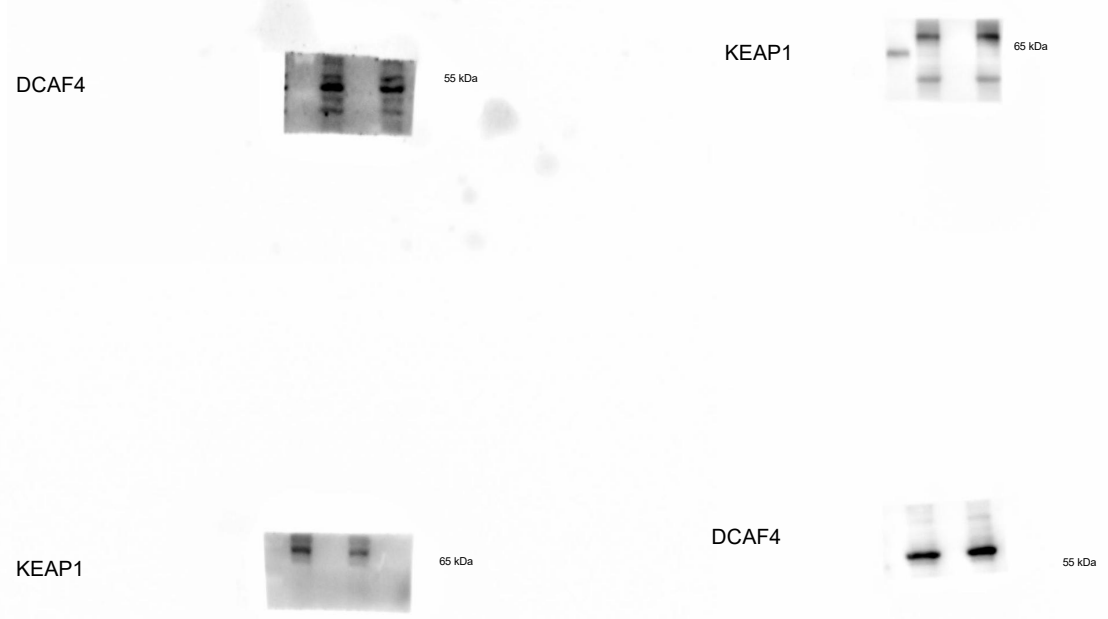

Figure4E

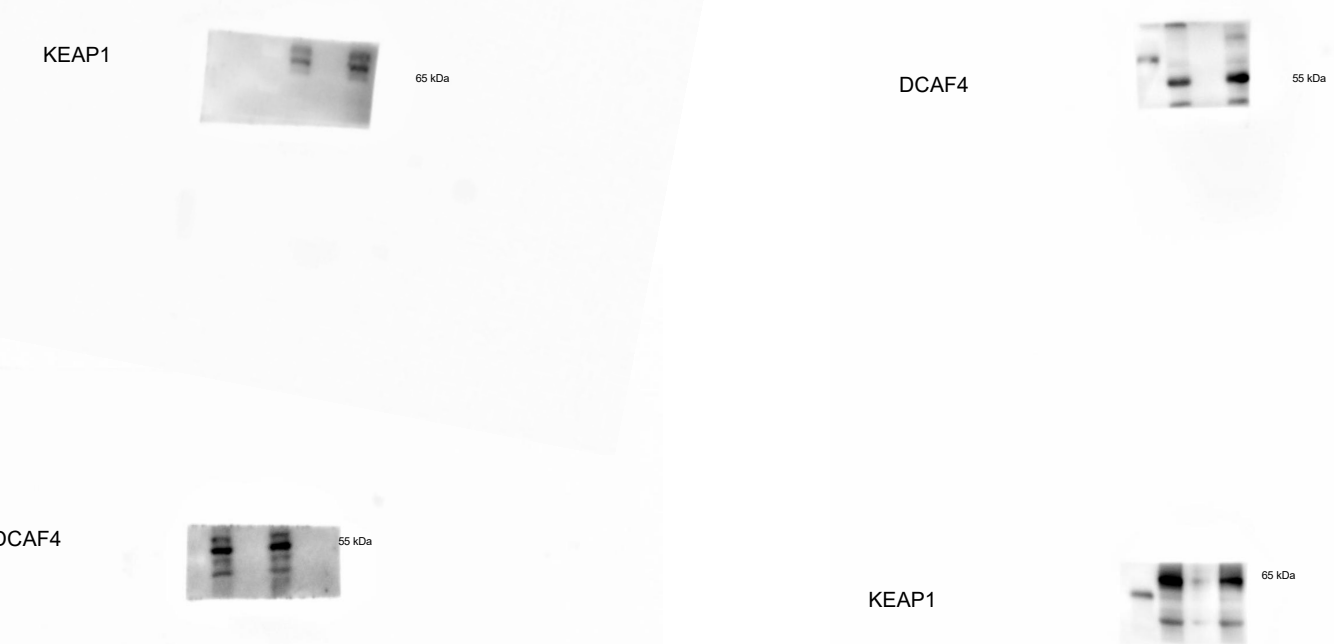

Figure4F

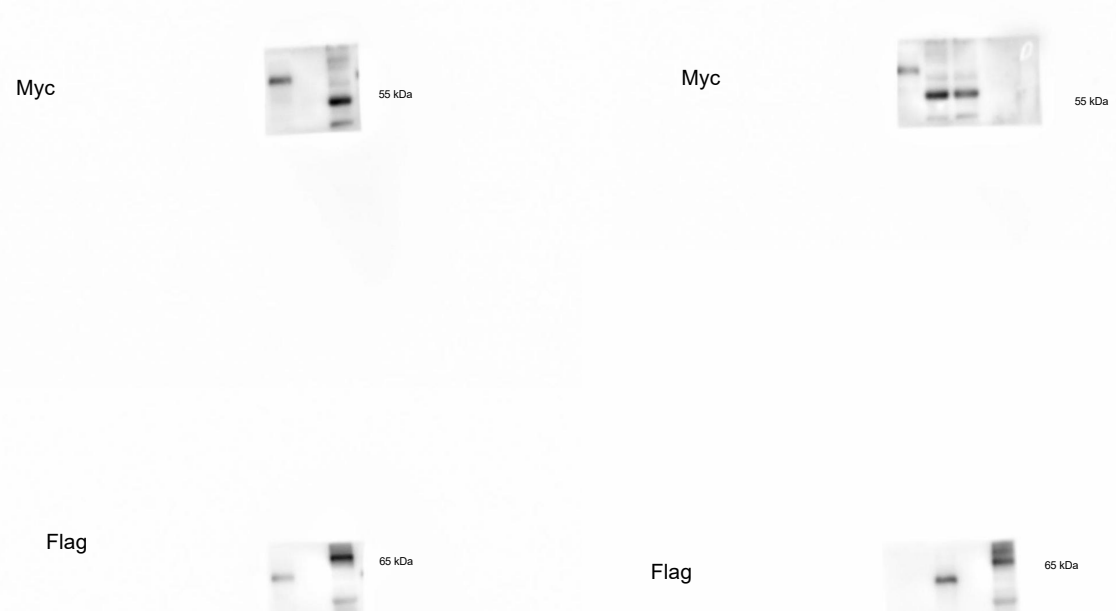

Figure4G

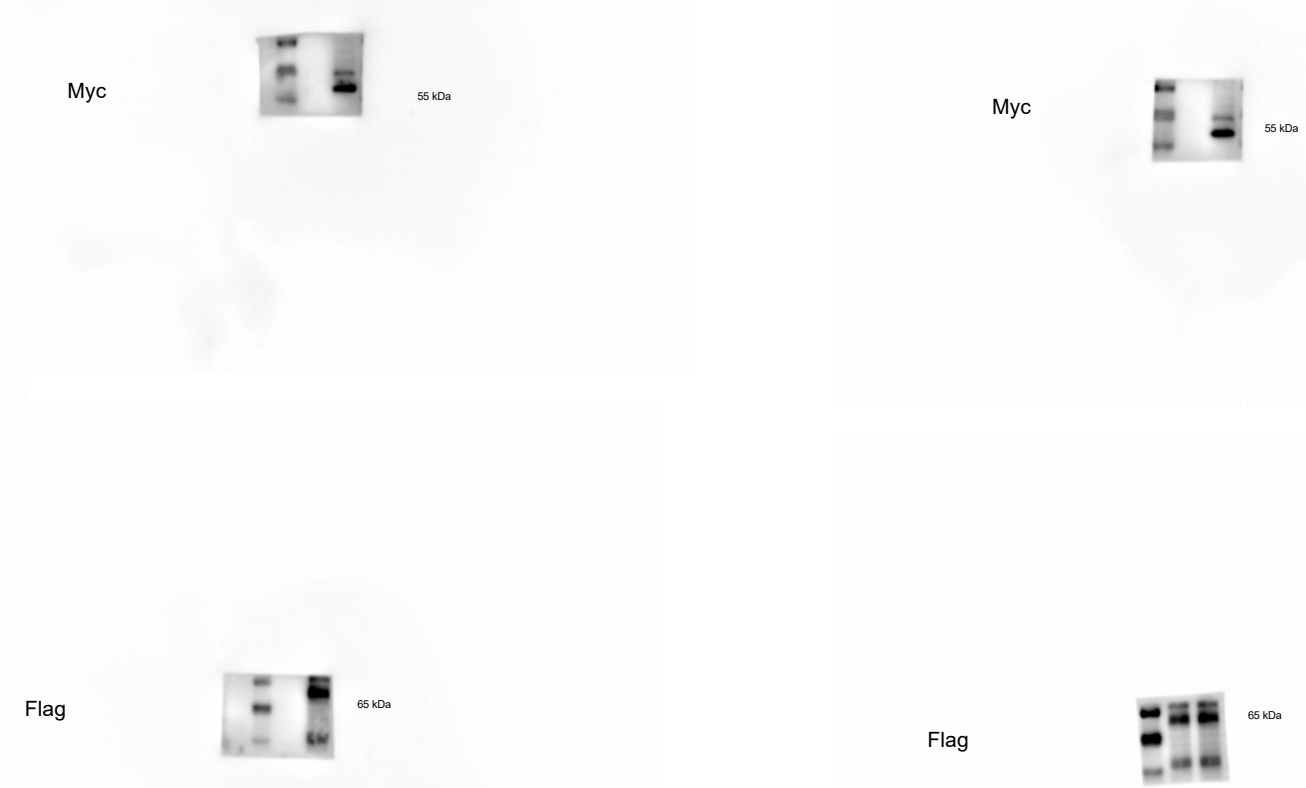

Figure4K

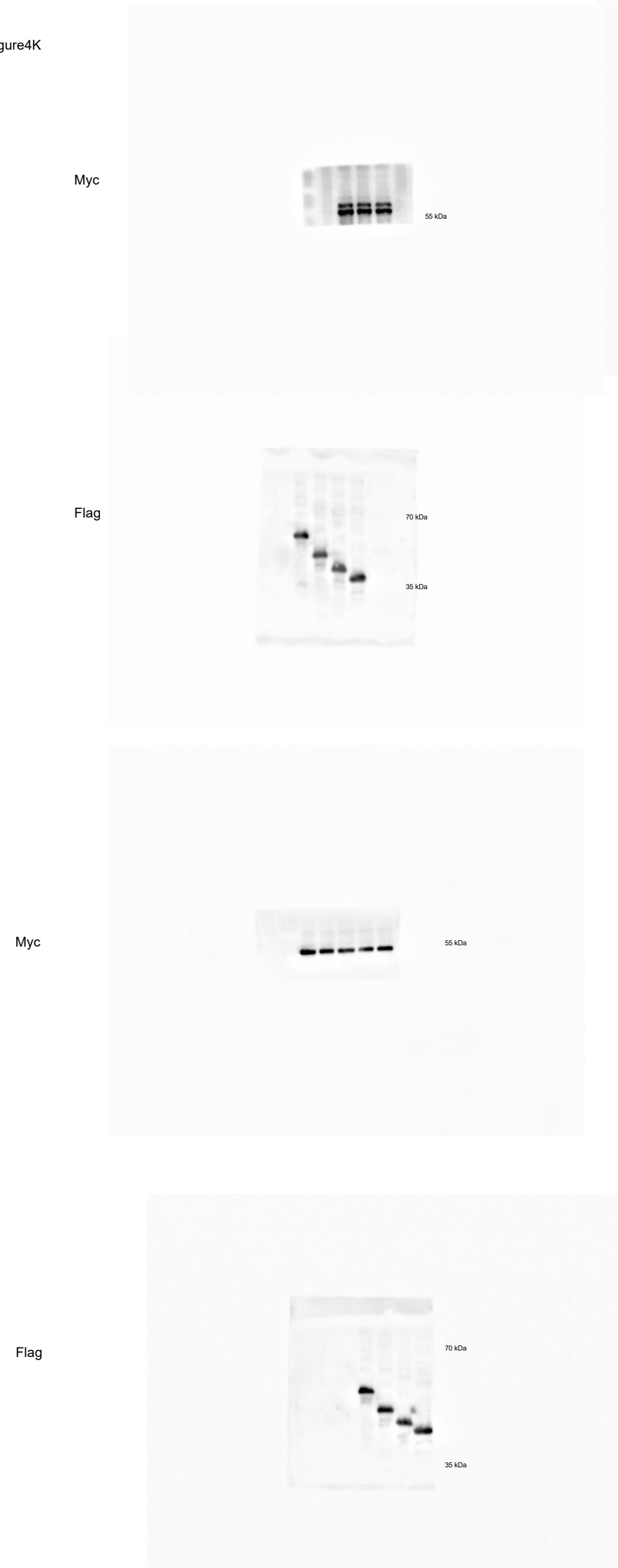

Figure4L

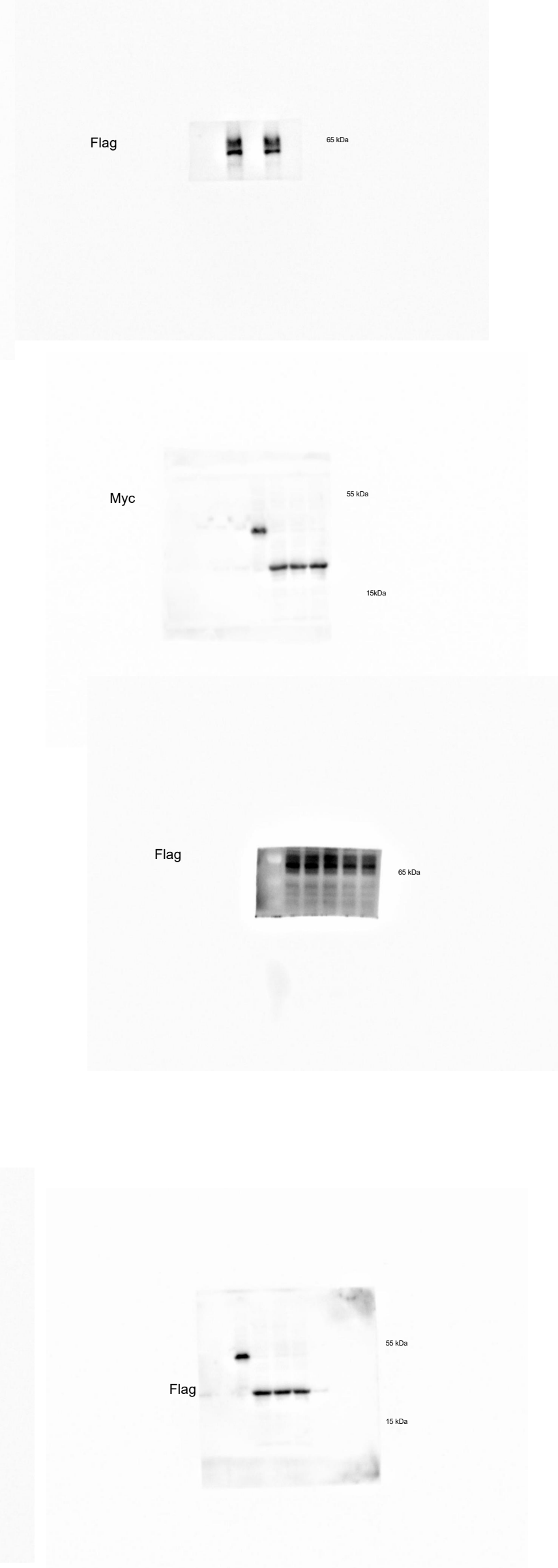

Figure4N

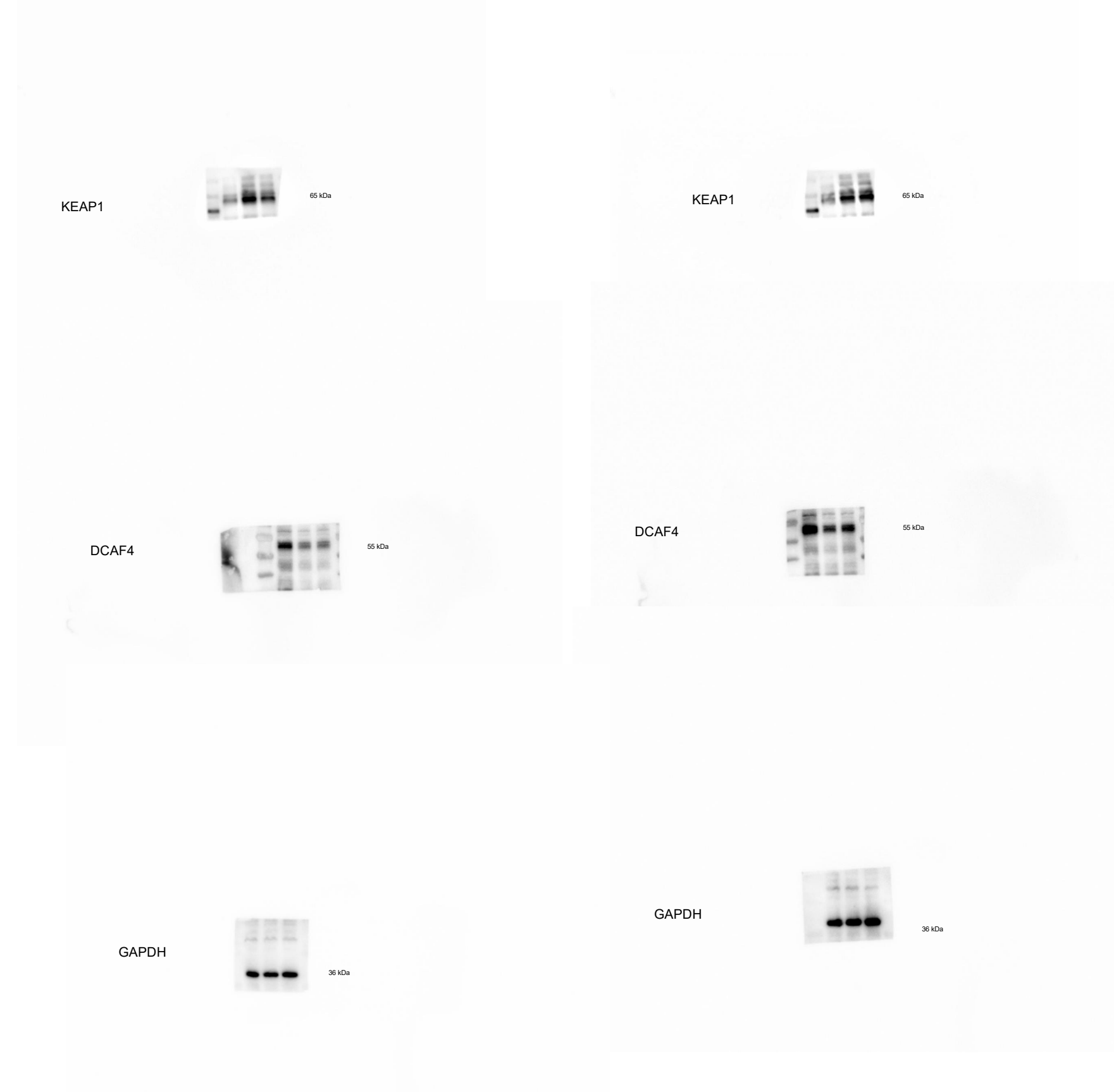

Figure4O

KEAP1

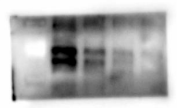

65 kDa

KEAP1

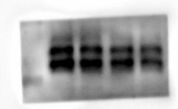

65 kDa

KEAP1

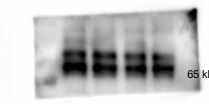

65 kDa

GAPDH

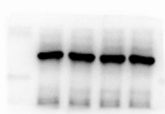

36 kDa

GAPDH

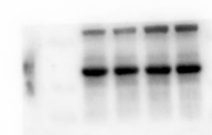

36 kDa

GAPDH

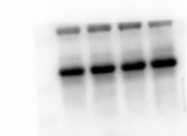

36 kDa

KEAP1

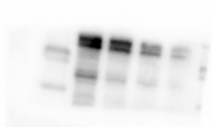

65 kDa

KEAP1

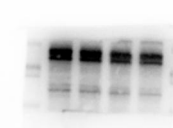

65 kDa

KEAP1

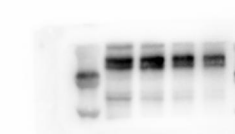

65 kDa

GAPDH

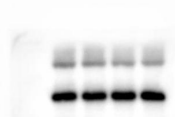

36 kDa

GAPDH

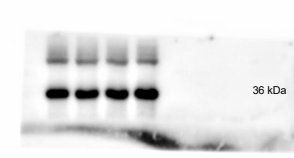

36 kDa

GAPDH

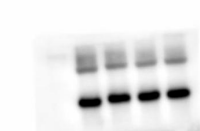

36 kDa

Figure4P

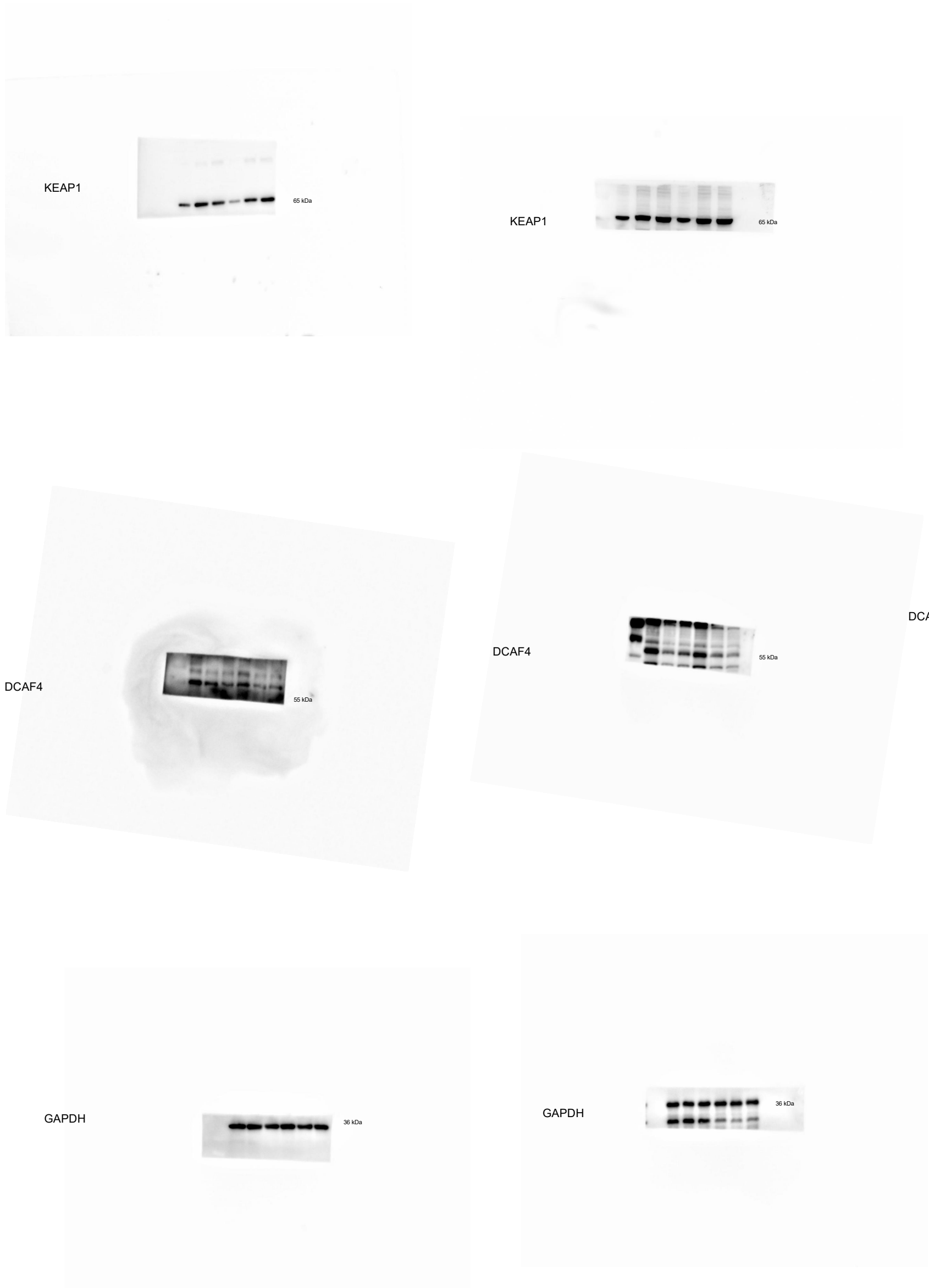

Figure4Q

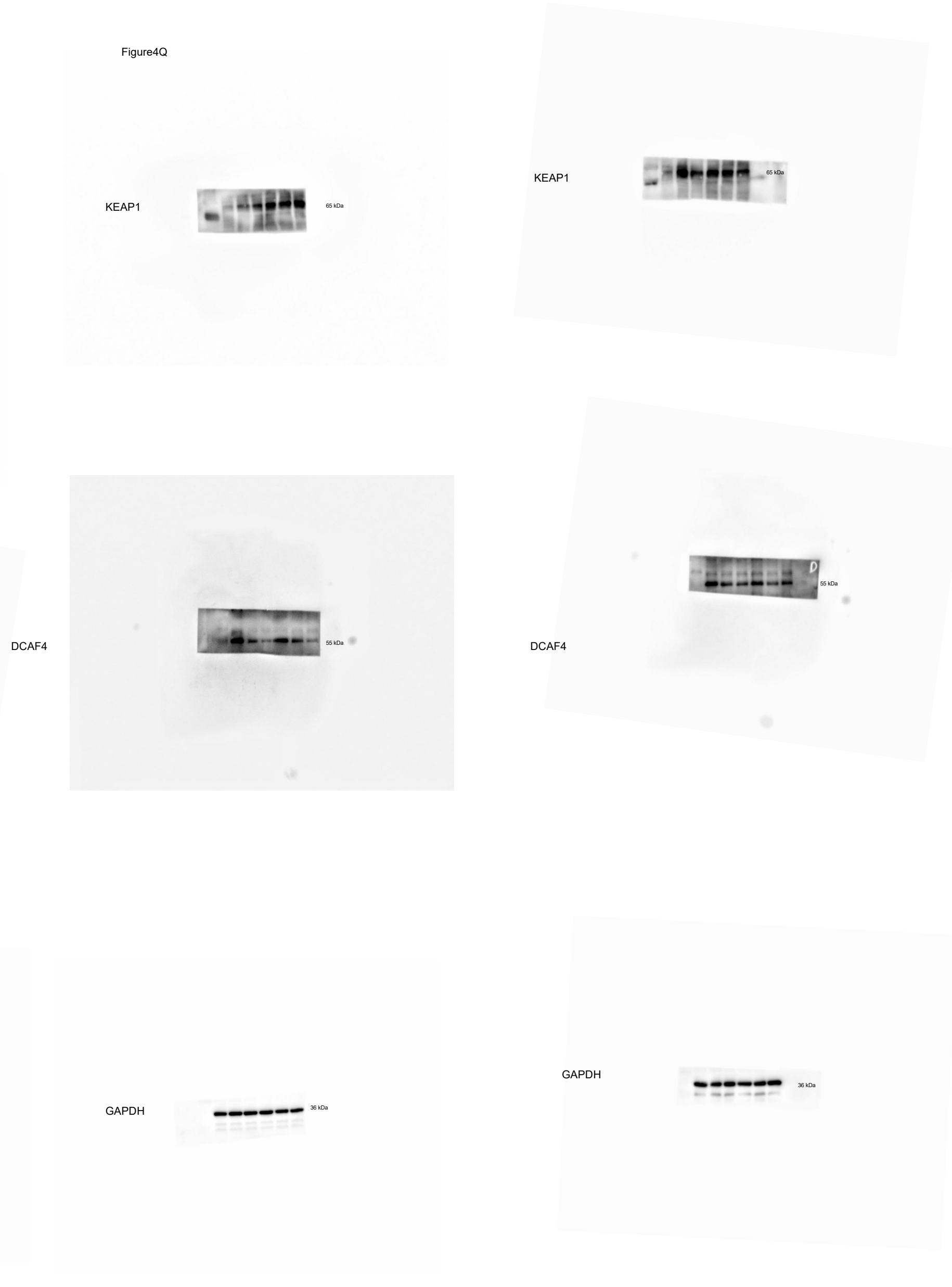

Figure5A-C

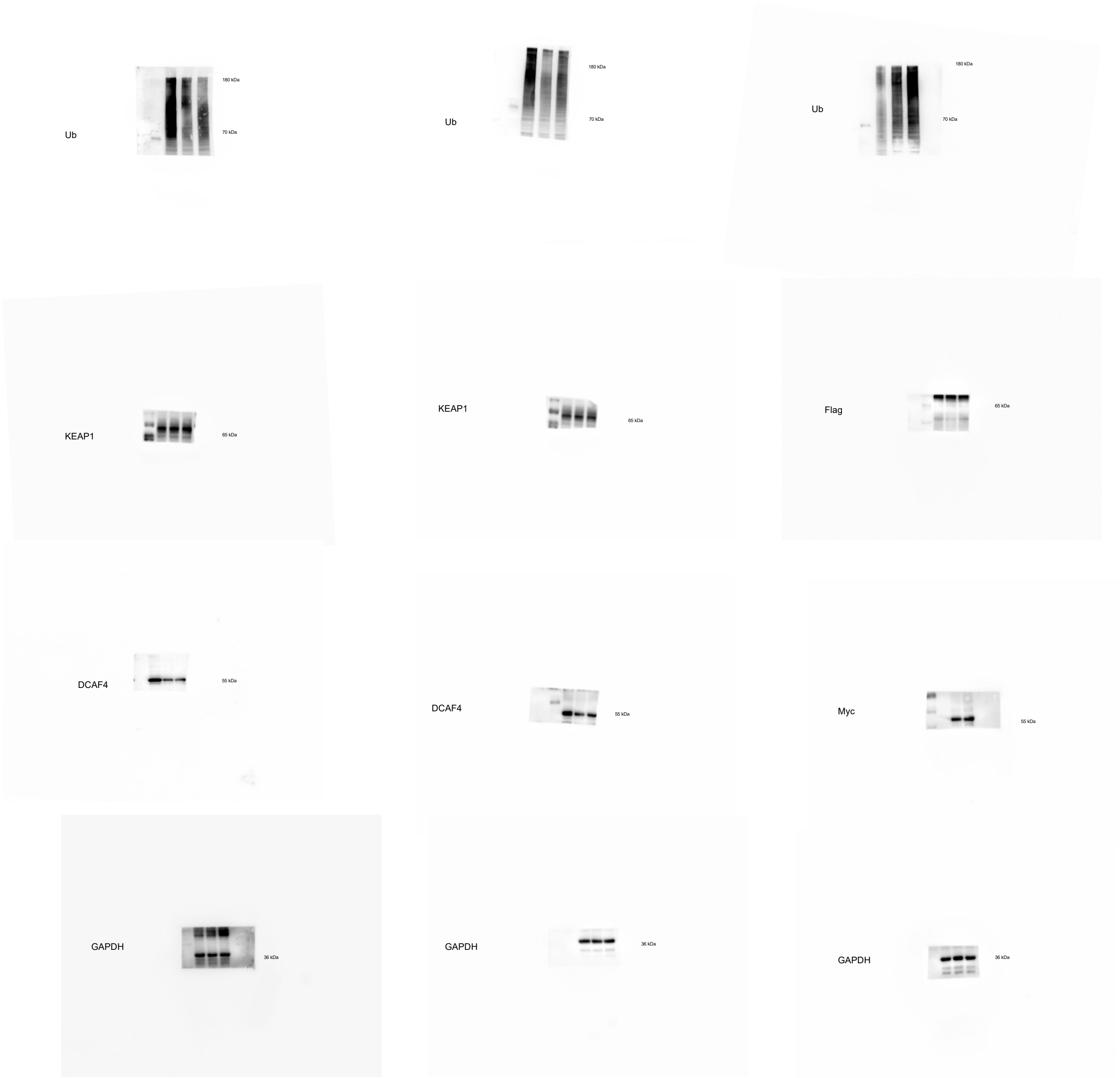
Ub
180 kDa
70 kDa
Ub
180 kDa
70 kDa
Ub
180 kDa
70 kDa
KEAP1
65 kDa
KEAP1
65 kDa
Flag
65 kDa
DCAF4
55 kDa
DCAF4
55 kDa
Myc
55 kDa
GAPDH
36 kDa
GAPDH
36 kDa
GAPDH
36 kDa

Figure5E-D

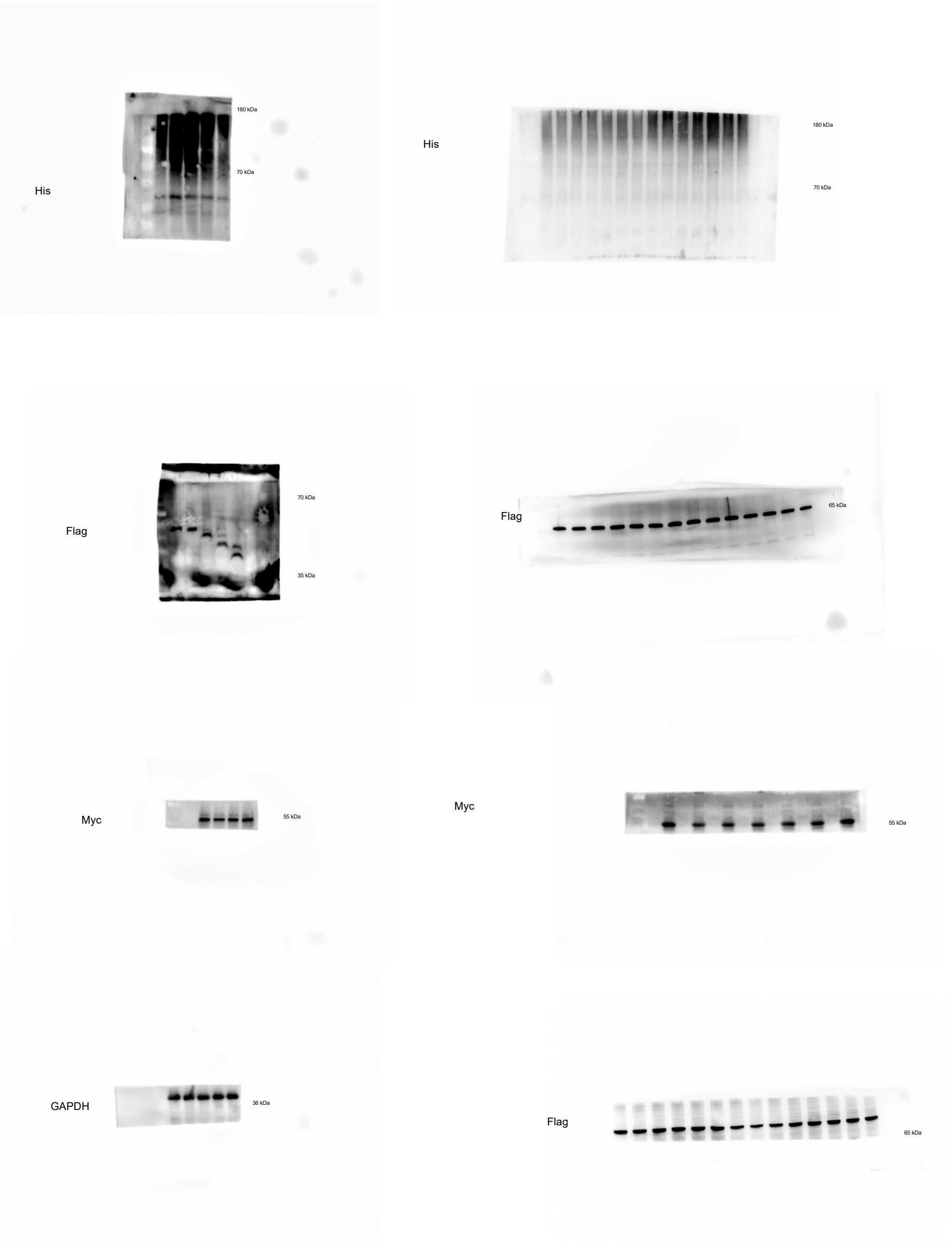
His
180 kDa
70 kDa
His
180 kDa
70 kDa
Flag
70 kDa
35 kDa
Flag
65 kDa
Myc
55 kDa
Myc
55 kDa
GAPDH
36 kDa
Flag
65 kDa

Figure5F-G

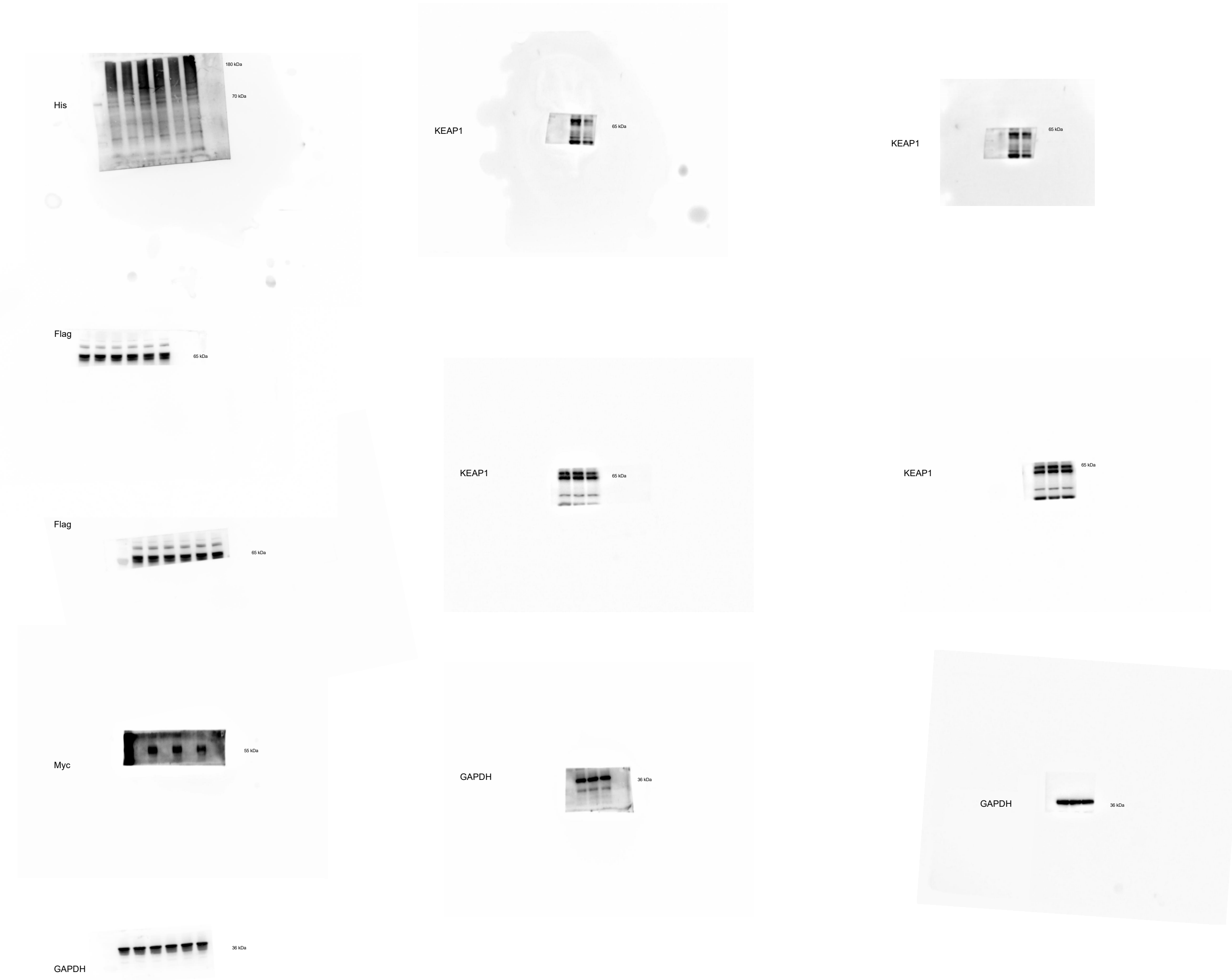
His
180 kDa
70 kDa
KEAP1
65 kDa
KEAP1
65 kDa
Flag
65 kDa
KEAP1
65 kDa
KEAP1
65 kDa
Flag
65 kDa
Myc
55 kDa
GAPDH
36 kDa
GAPDH
36 kDa
GAPDH
36 kDa

Figure5J-L

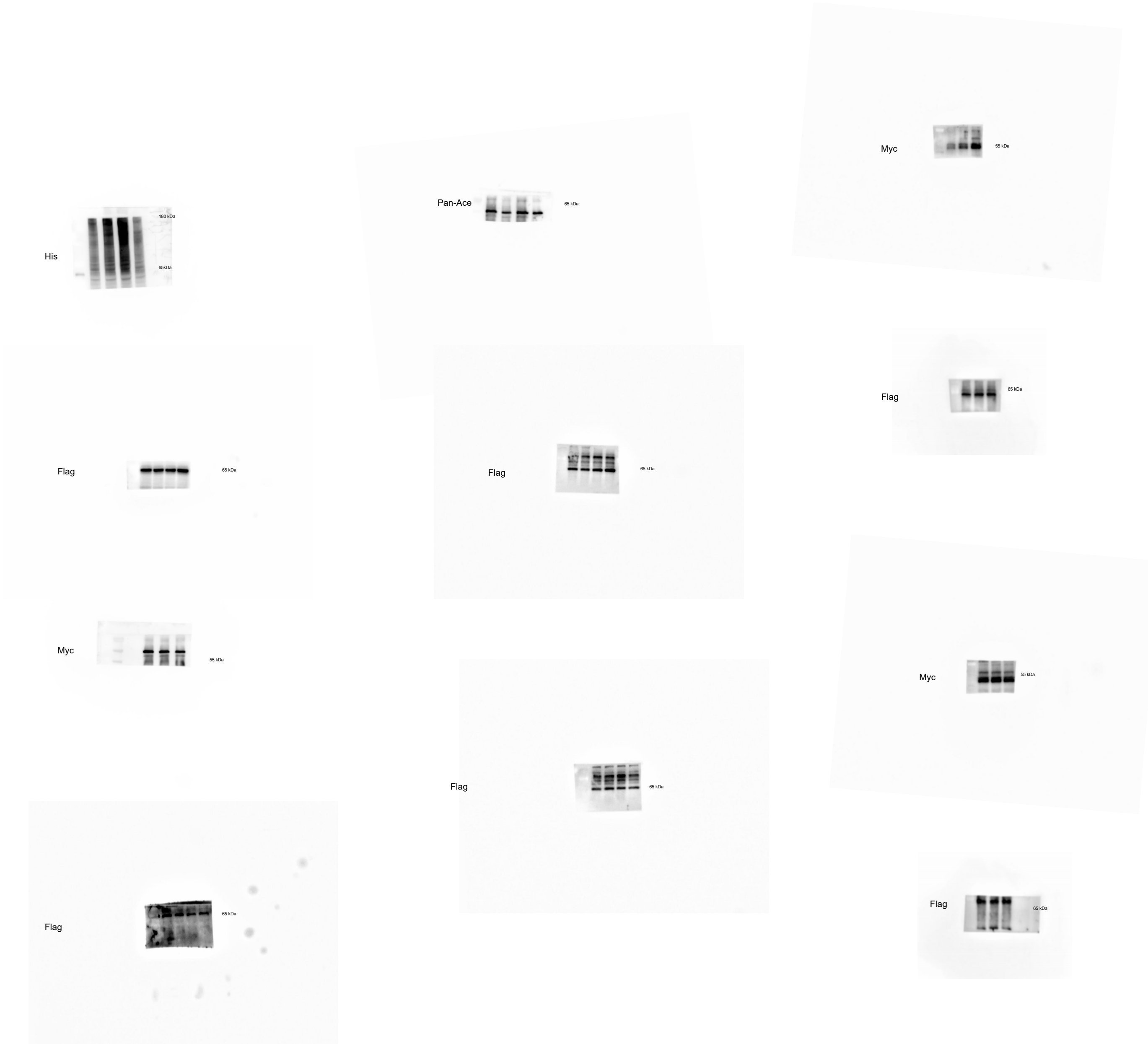

His
180 kDa
65kDa
Flag
65 kDa
Myc
55 kDa
Flag
65 kDa
Pan-Ace
65 kDa
Flag
65 kDa
Flag
65 kDa
Myc
55 kDa
Flag
65 kDa
Myc
55 kDa
Flag
65 kDa

Figure6D-G

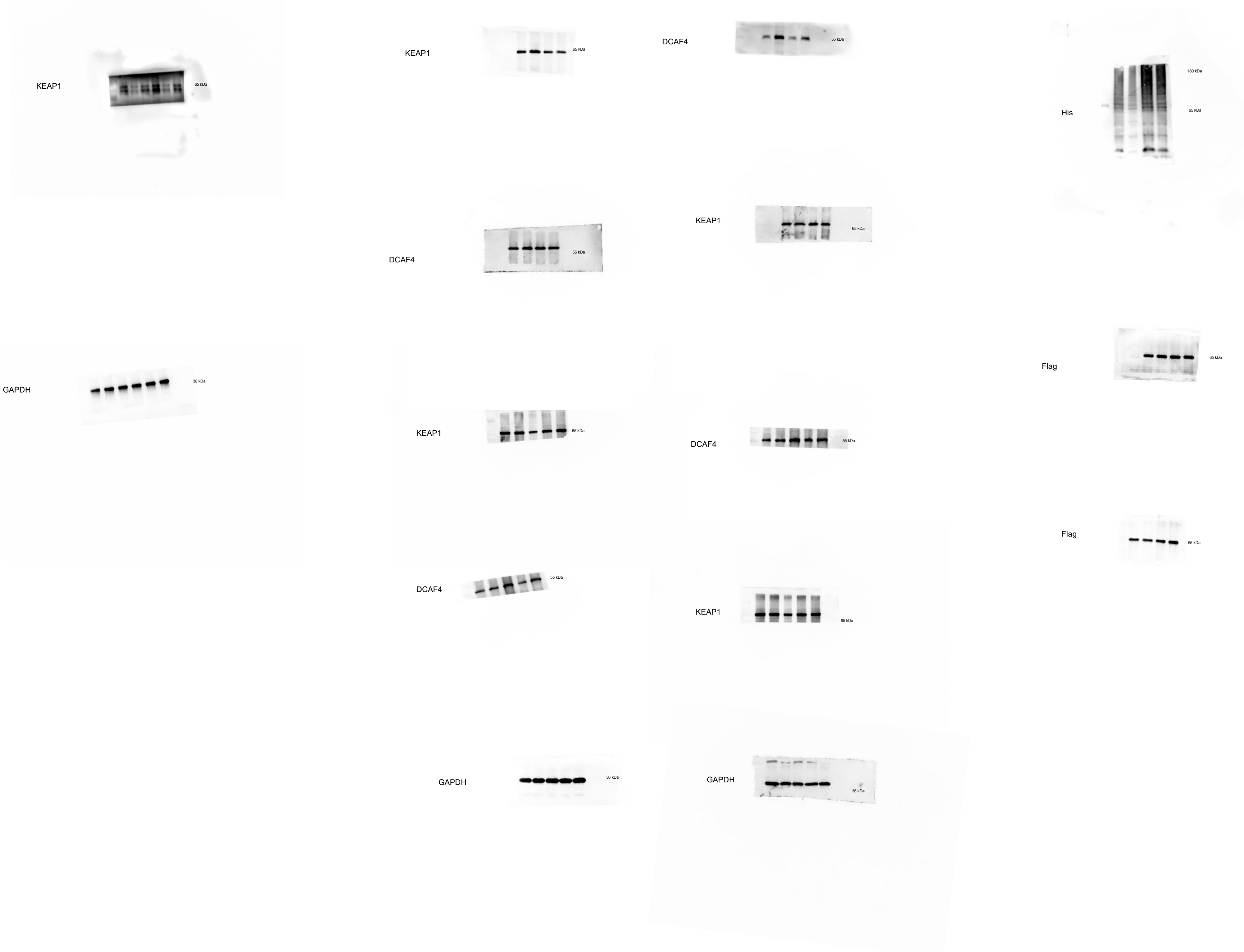
KEAP1
65 kDa
GAPDH
36 kDa
KEAP1
65 kDa
DCAF4
55 kDa
KEAP1
65 kDa
DCAF4
55 kDa
GAPDH
36 kDa
DCAF4
55 kDa
KEAP1
65 kDa
DCAF4
55 kDa
KEAP1
65 kDa
GAPDH
36 kDa
His
180 kDa
65 kDa
Flag
65 kDa
Flag
65 kDa

Figure6H

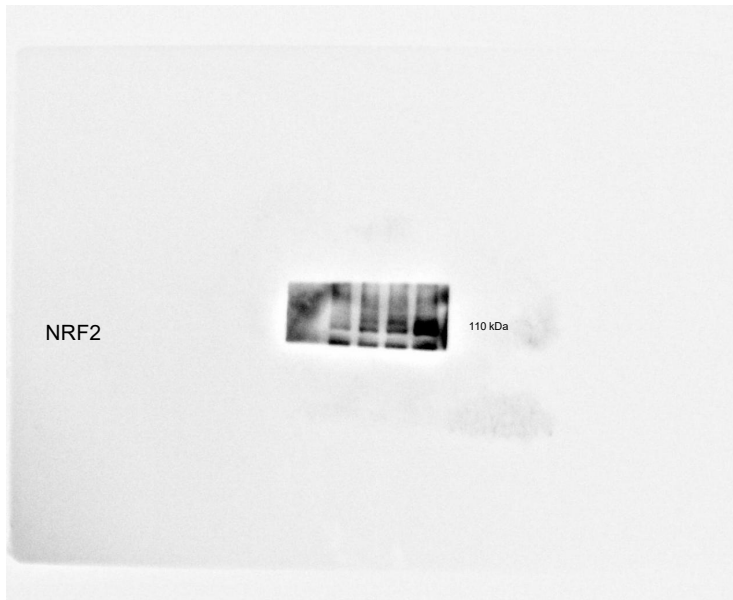

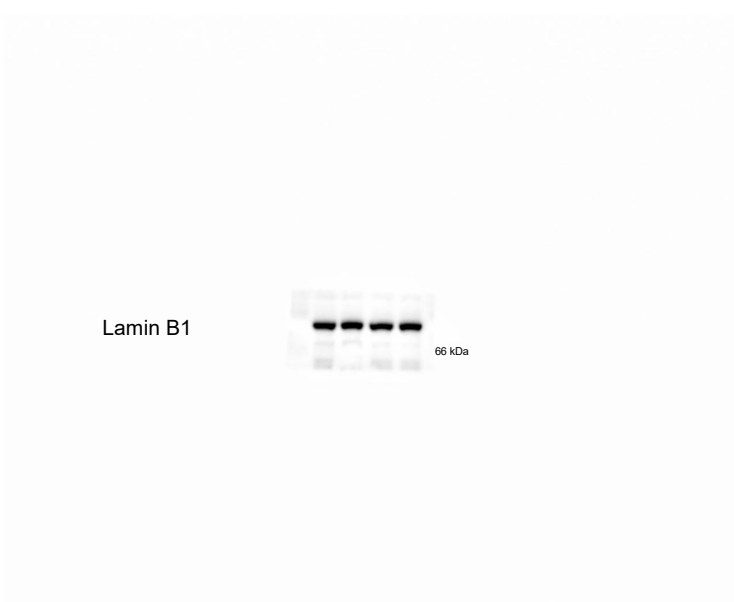

Figure7E-F

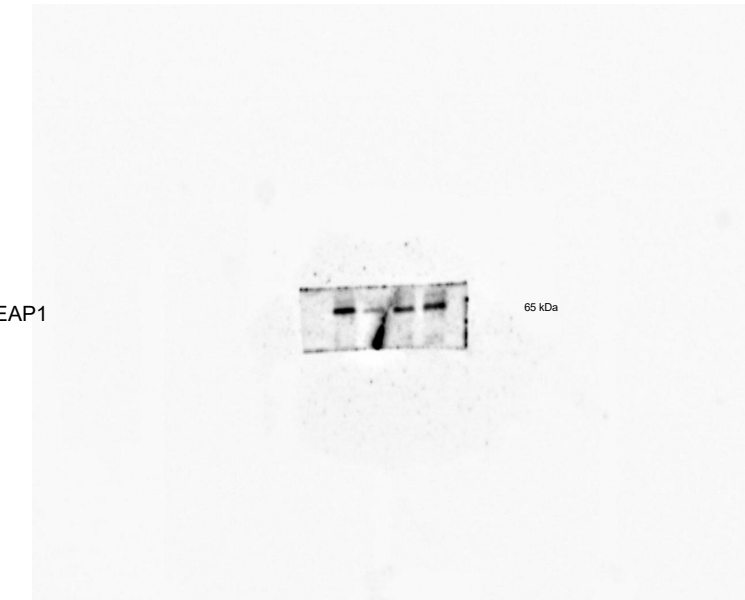

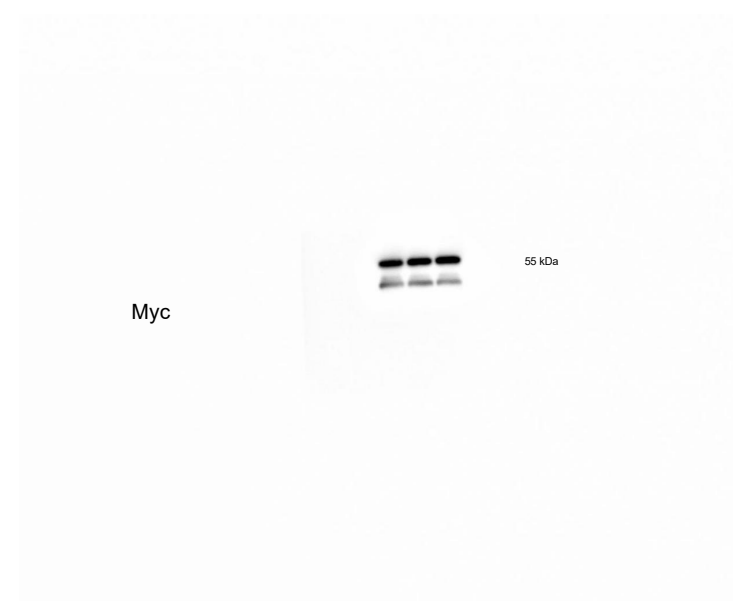

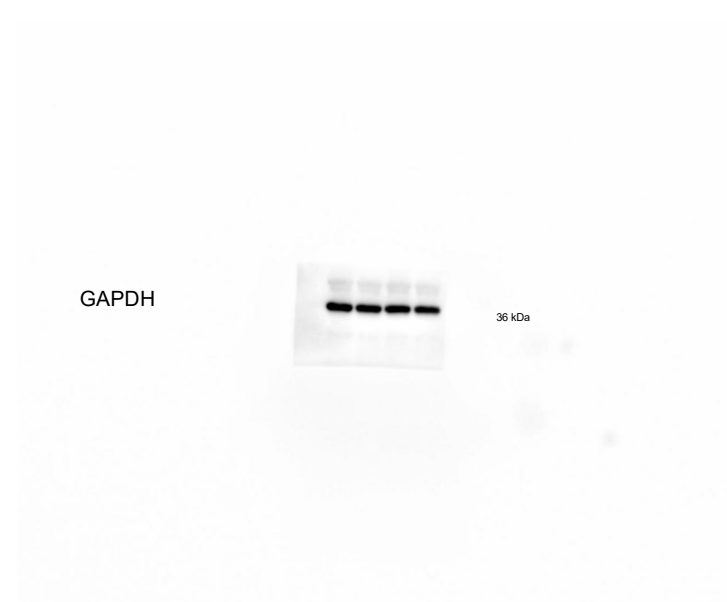

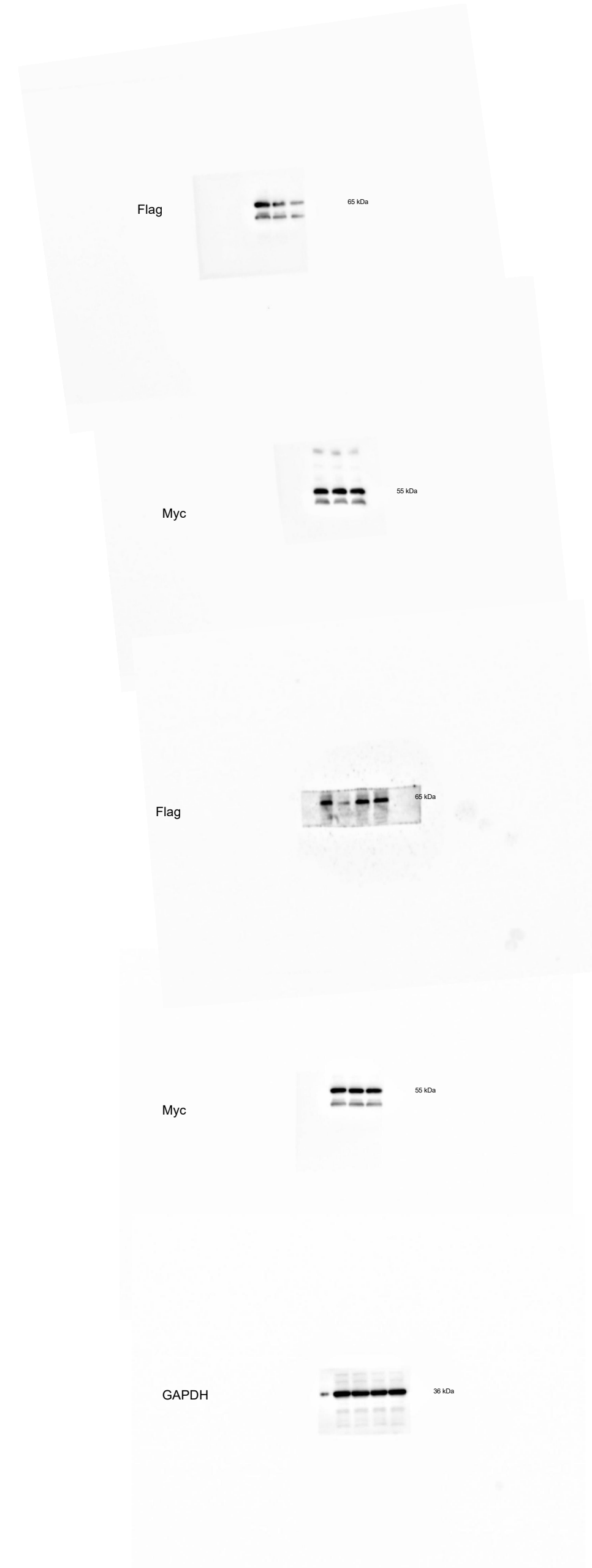

FigureS1M

DCAF4

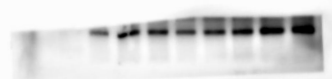

55 kDa

GAPDH

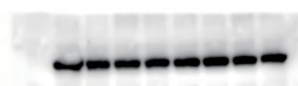

36 kDa

FigureS1S

DCAF4

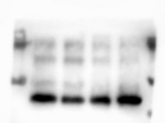

55 kDa

GAPDH

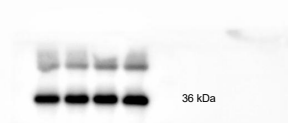

36 kDa

FigureS1T

DCAF4

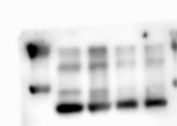

55 kDa

GAPDH

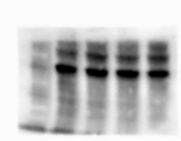

36 kDa

FigureS1V

DCAF4

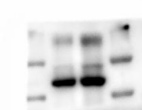

55 kDa

GAPDH

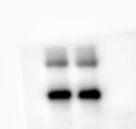

36 kDa

FigureS3G

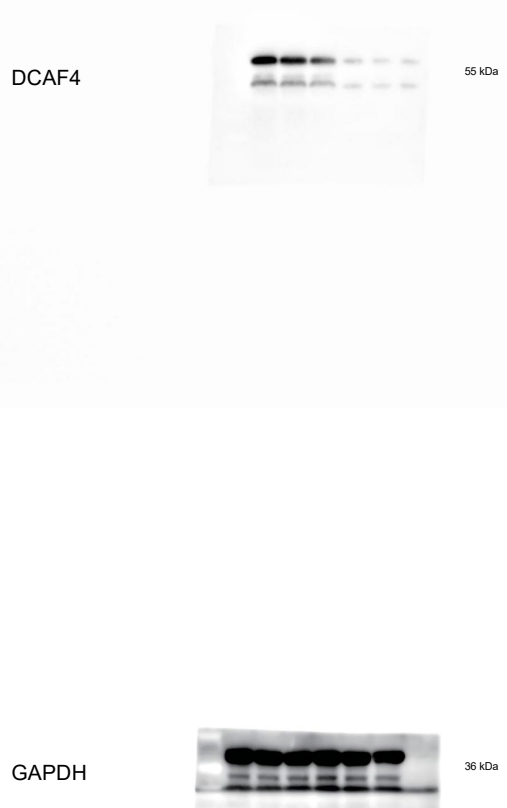

FigureS4A

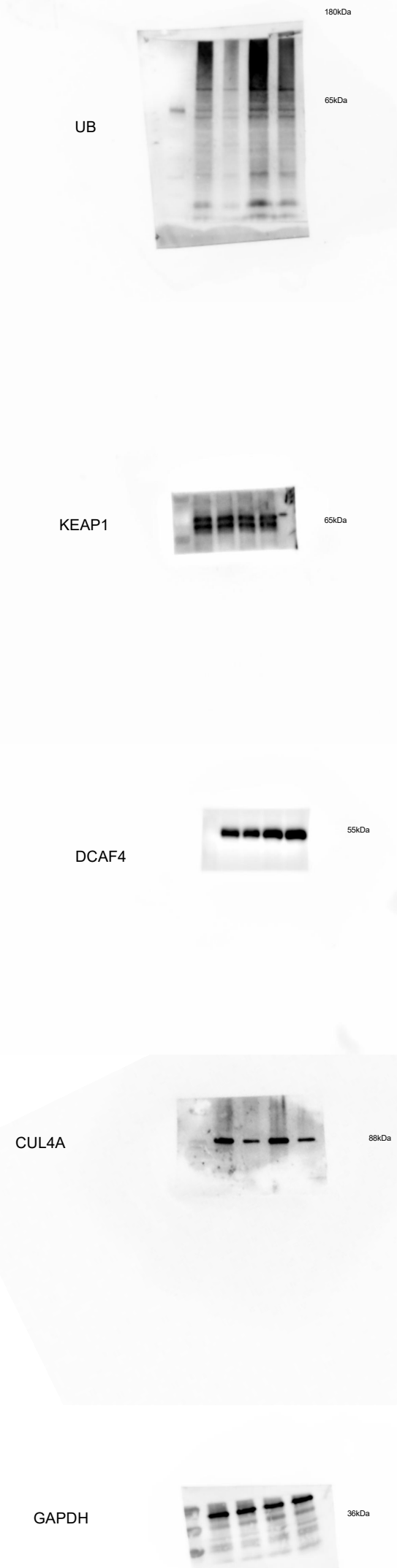

FigureS4B

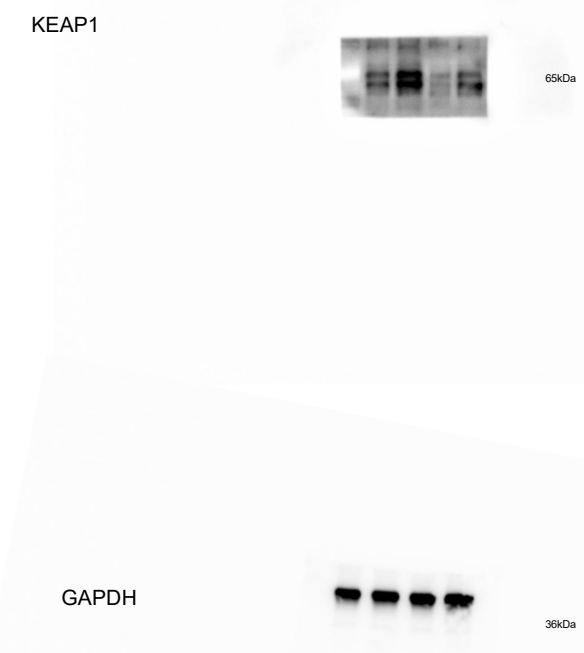

FigureS5A

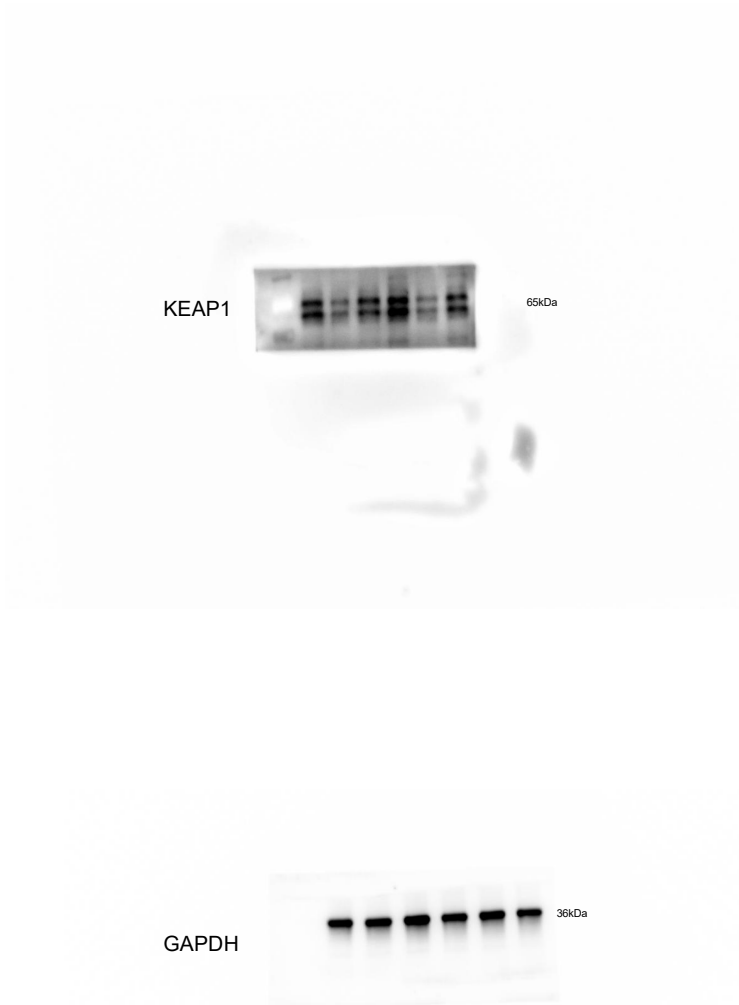

FigureS5K-L

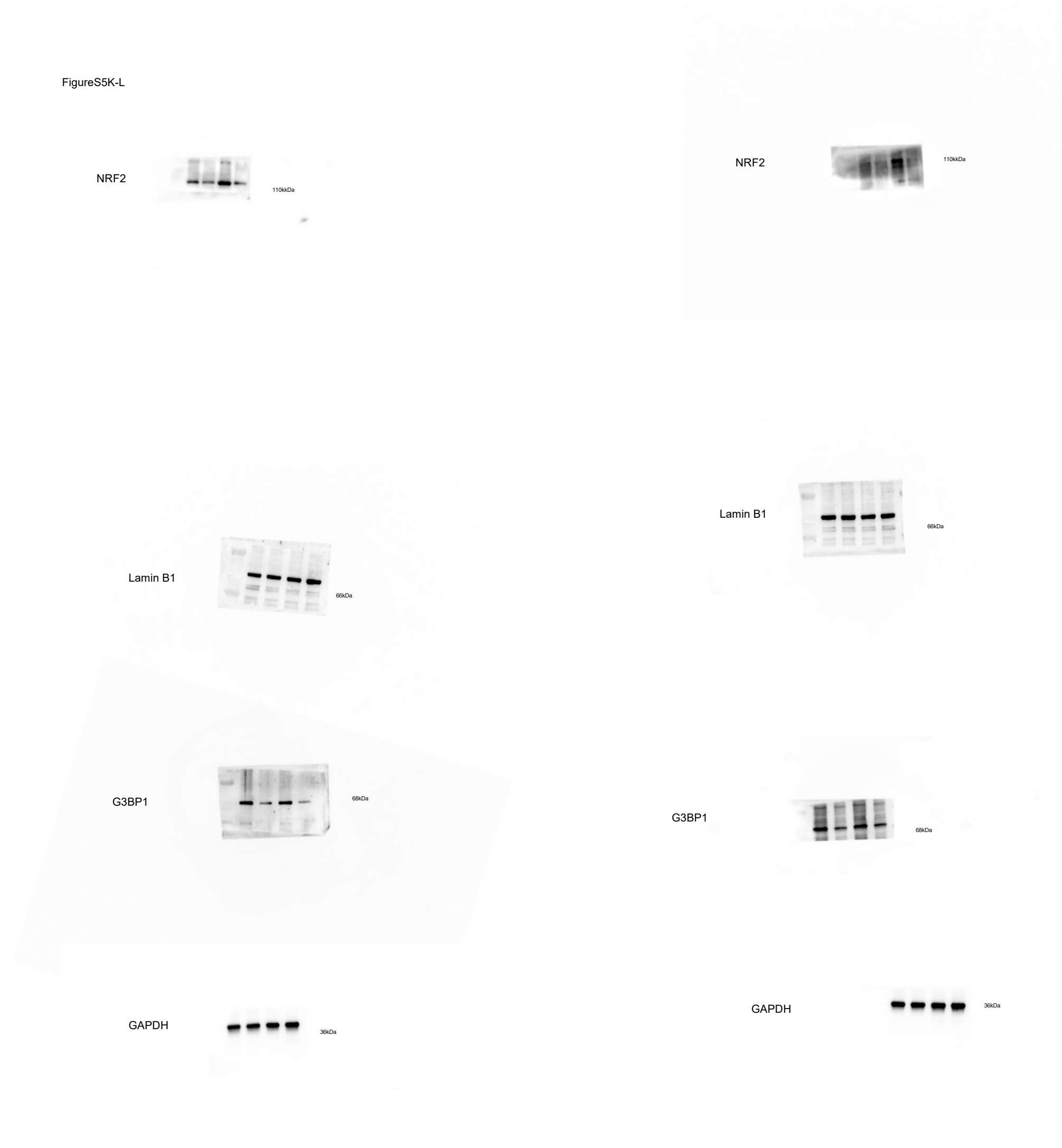

FigureS5I-J

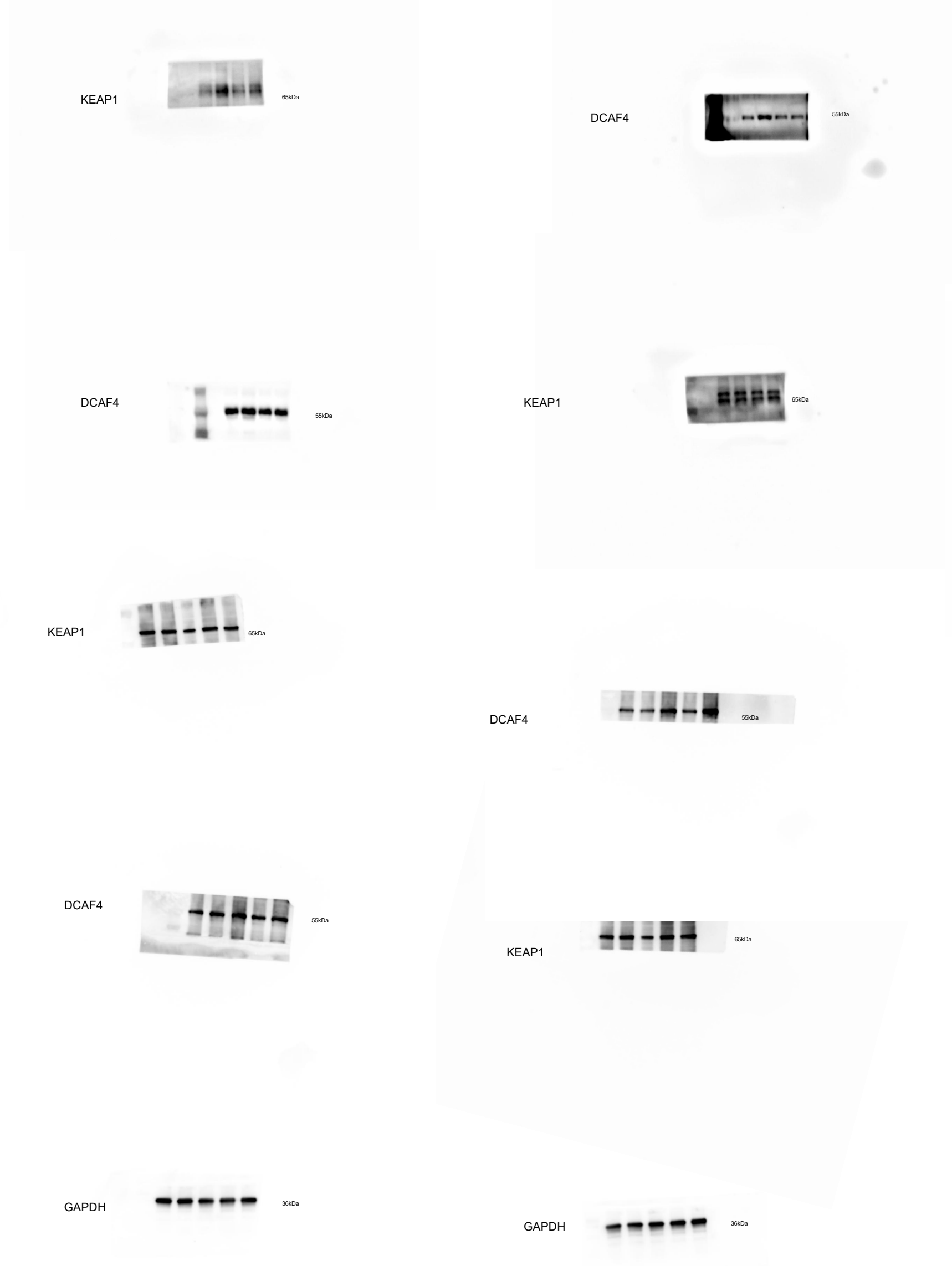

FigureS6D-E

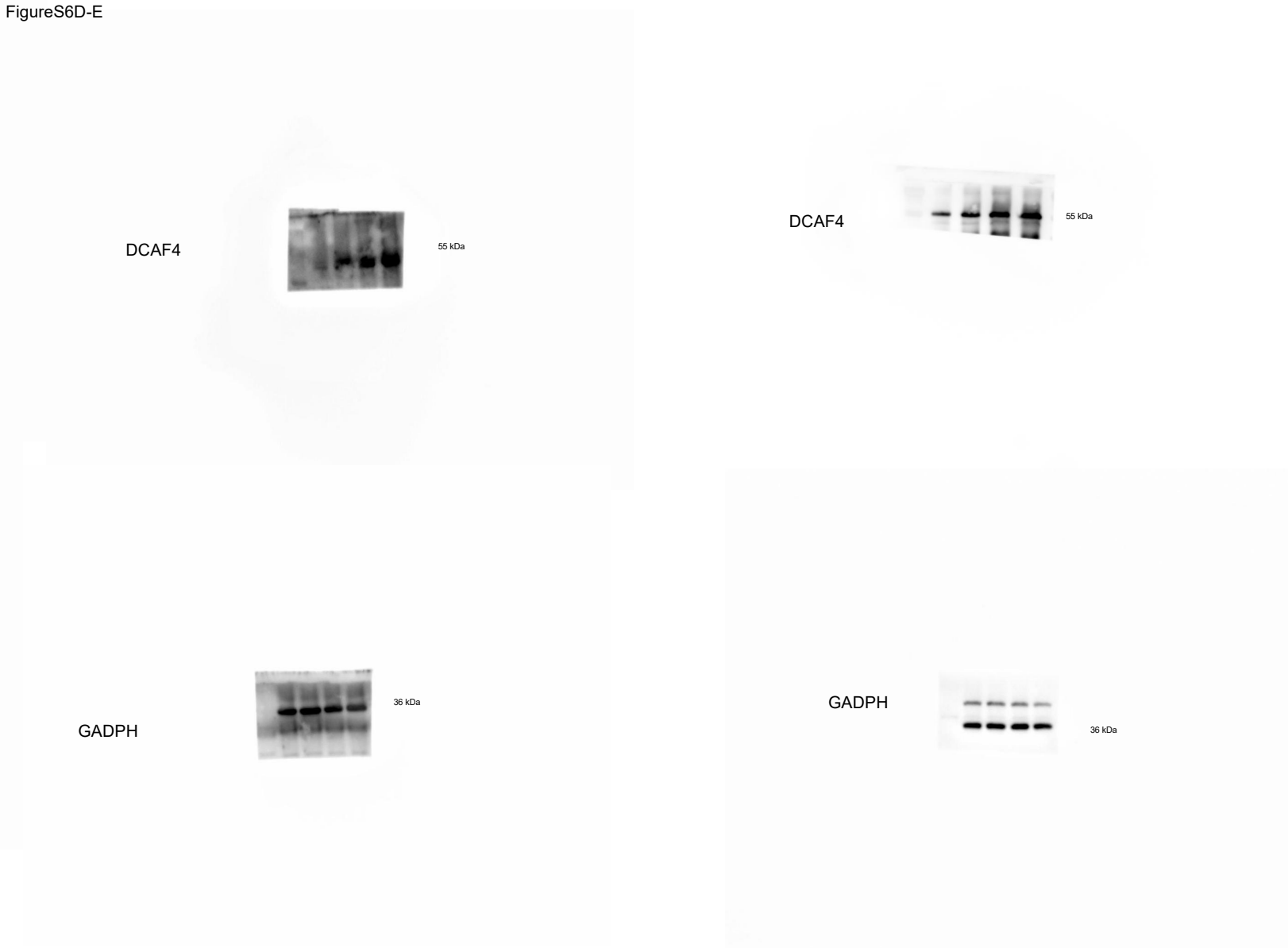

FigureS6I

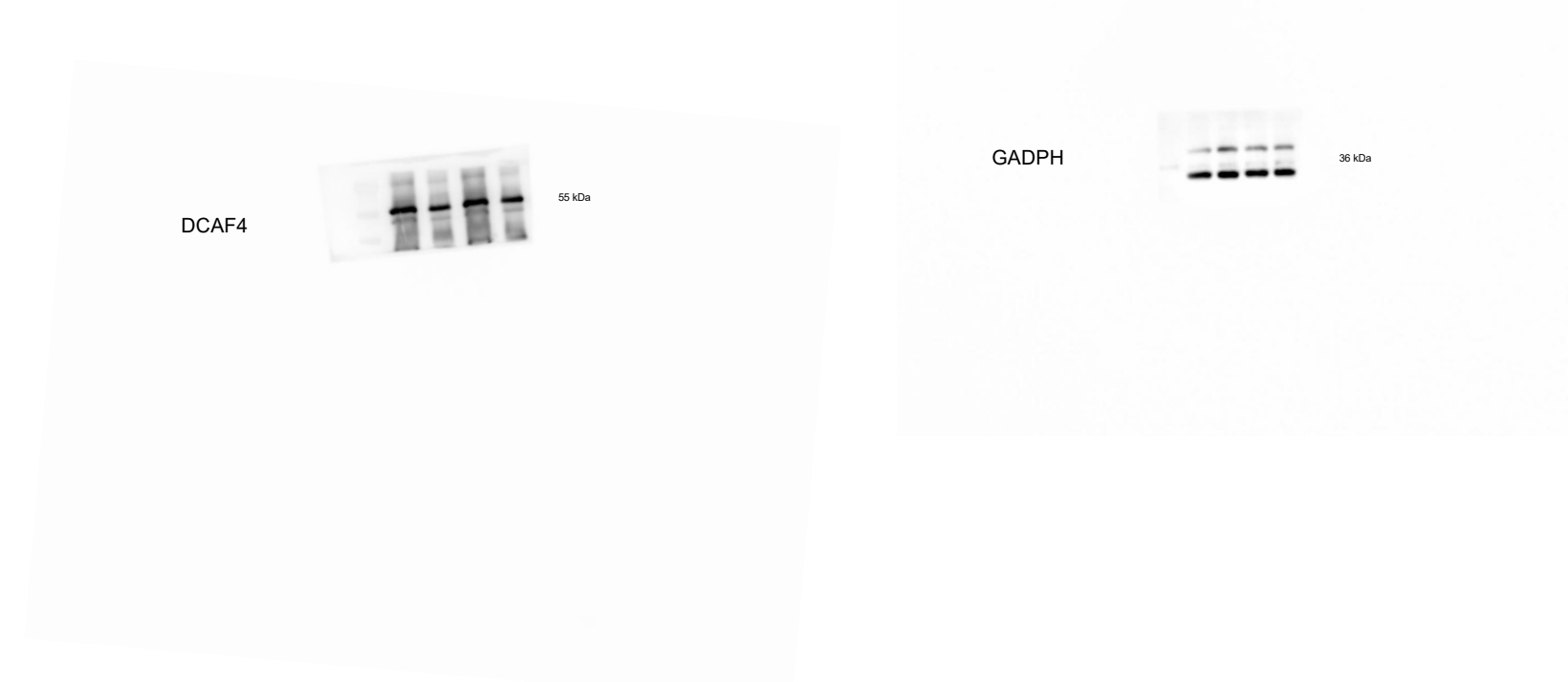

Supplement: Supplementary file 1 [file 41419_2026_9095_MOESM10_ESM.pdf]
