# Supplementary material for: CRISPR/Cas9 screen identifies DCAF4 as a novel protector of hepatocellular carcinoma against brachytherapy via stress granule-dependent NRF2 activation
Source: Cell Death Dis. 2026 Jul 14;17(1):818. doi: 10.1038/s41419-026-09095-0 (PMC13594110; doi:10.1038/s41419-026-09095-0)
Supplement: Supplementary file 2 [file 41419_2026_9095_MOESM1_ESM.pdf]

Supplementary materials and methods

Pooled genome-wide CRISPR screening

The human CRISPR knockout pooled library (Brunello)(Addgene #73179) was obtained from Nanjing Coris Biological Co., Ltd. (Nanjing, China). Amplification of the sgRNAs and packaging of the lentivirus were conducted according to the protocol established by Zhang's Lab. The Huh7 HCC cell line was engineered to stably express Cas9 through lentiviral transduction. Briefly, 1.5×10^7 cells were transduced with the Brunello sgRNA library at a multiplicity of infection (MOI) of 0.3 in culture medium supplemented with 8 μg/mL polybrene. The cell–virus mixture was centrifuged at 1,000 × g for 2 h at 30 °C, followed by overnight incubation at 37 °C with 5% CO2. After transduction, cells were selected with 2 μg/mL puromycin for 3 days and then allowed to recover and expand for an additional 4 days. In the in vitro screening phase, one group of cells was collected at baseline (T0) prior to intervention, while the other two groups were exposed to either tert-butyl hydroperoxide (TBHP) or vehicle control for 14 days. In the in vivo experiments, transduced cells were implanted subcutaneously into the right flank of mice, followed by BT treatment initiation 7 days post-injection. Here, one cohort was harvested at baseline (T0), and the remaining groups received either BT or control treatment for a duration of 14 days. Subsequently, cells were collected and genomic DNA was extracted using manufacturer-recommended protocols. The sgRNA cassettes were amplified via PCR and submitted for Illumina sequencing (Illumina, San Diego, USA), which was performed by Genewiz (Suzhou, China). Enrichment of sgRNAs and statistical analyses were performed with MAGeCK by bioinformatics specialists at Genewiz.

Bioinformatics analysis

Clinical sample analysis was performed using the TCGA, GSE136247, GSE87630, GSE39791 and GSE36376 datasets. mRNA sequencing data, along with clinical information for Liver Hepatocellular Carcinoma (LIHC) was acquired from the Cancer

Genome Atlas (TCGA) (https://portal.gdc.cancer.gov) and the Gene Expression Omnibus (GEO) (https://www.ncbi.nlm.nih.gov/geo). ssGSEA was performed to calculate enrichment scores for HCC tissues in the GSE9843, GSE25097, GSE63898, and ICGC datasets. Samples with scores in the top 20% were defined as oxidative stress-active. Differentially expressed genes (DEGs) were identified between oxidative stress-active and inactive samples within each dataset, and the common DEGs across all four datasets were obtained by taking the intersection. We integrated and analyzed publicly available single-cell datasets for HCC to examine DCAF4 mRNA expression dynamics. Individual cells were selected for further analysis based on the following criteria: a mitochondrial gene proportion of ≤10% of total unique molecular identifiers (UMIs) and UMI counts between 3,000 and 40,000. Subsequently, gene expression levels (UMI + 1) were normalized and log2-transformed.

Cell and cell culture

We obtained the human immortalized hepatocyte cell line THLE-2, human HCC cell lines (Huh7, Hep3B, and HCCLM3) and mouse cell line Hepa1-6 from the Cell Bank of the Chinese Academy of Sciences. All cell lines were cultured in Dulbecco's Modified Eagle Medium (DMEM; Gibco, USA), supplemented with 10% fetal bovine serum and 1% penicillin/streptomycin, and maintained at 37 °C in a humidified incubator with 5% CO_2.

Western blotting

Cellular proteins were extracted with RIPA buffer, subjected to SDS-PAGE, and electrotransferred to PVDF membranes. After blocking, the membranes were probed with primary antibodies at 4°C overnight, followed by an HRP-conjugated secondary antibody. Immunoreactive bands were detected using an Enhanced Chemiluminescence (ECL) kit (Thermo Fisher, USA). Antibody details are provided in Table S1.

RNA extraction and quantitative real-time polymerase chain reaction (qRT-PCR)

We utilized the FastPure® Cell/Tissue Total RNA Isolation Kit V2 (Vazyme, China) to extract total RNA from cells and samples as per the provided instructions by the manufacturer. By employing a reverse transcription kit (Vazyme, China), the RNA was transcribed in reverse into cDNA. The specific sequences of each primer are presented in Table S2. In order to establish normalized mRNA expression levels, we used GAPDH as a reference.

Cell transfection

Briefly, 1×10^5 cells per well were seeded in six-well plates and cultured in 2 mL of medium for 24 hours. The medium was then replaced with 1 mL containing an appropriate amount of virus and 40 μL of polybrene (Sigma-Aldrich, USA). After 12–16 hours of incubation, the cells were returned to normal medium and selected with puromycin. For siRNA or plasmid transfection, Lipofectamine 3000 (Invitrogen, USA) was used according to the manufacturer's instructions. The sequences of shRNAs and siRNAs are listed in Table S3. Transfection efficiency was evaluated by qRT-PCR or western blotting.

Plasmid constructs

The plasmids encoding wild-type DCAF4, mutant KEAP1, wild-type KEAP1, wild-type ubiquitin and mutant ubiquitin were synthesized by Transheep (Shanghai, China). The DNA sequences of these constructs were verified by DNA sequencing at Transheep Biological Technology Co. (Shanghai, China).

Cell proliferation assay

Cell proliferation was assayed using a CCK-8 kit (RiboBio, China). Briefly, Huh7 and HCCLM3 cells were plated in 96-well plates (1,000 cells/well) and grouped accordingly. After adding 10 μL of CCK-8 solution to each well, the optical density at 450 nm was recorded at 0, 24, 48, and 72 hours using a microplate reader (Synergy, USA).

Colony formation assay

HCC cells in different groups were seeded in a 6-well plate (500 cells/well) and cultured in 4mL of the medium at 37°C for 2 weeks. The proliferating colonies were then fixed with 4% paraformaldehyde (Beyotime, China) and stained with crystal violet (Beyotime, China). Finally, the visible colonies were photographed and counted.

Cell apoptosis assay

Apoptotic cells were quantified using an Annexin V-FITC/PI Apoptosis Detection Kit (Vazyme Biotech, China) and analyzed on a Cytoflex flow cytometer (Beckman, China), following the manufacturer's protocol. Data analysis was performed with FlowJo V10 software.

Immunoprecipitation coupled with mass spectrometry (IP/MS)

Proteins were extracted from transfected cells and immunoprecipitated using primary antibodies on Protein A/G-agarose beads. The immunoprecipitates were analyzed by mass spectrometry. The experiment and preliminary analysis were conducted by Hangzhou Cosmos Wisdom Biotech Co., Ltd.

RNA sequencing

Transcriptome analysis was conducted to profile the effects of DCAF4 silencing. Total RNA from DCAF4-knockdown and control Huh7 cells, isolated with TRIzol, was used for RNA sequencing, which was carried out by Berry Genomics (Beijing, China).

Co-Immunoprecipitation (Co-IP) assay

HEK293T or HCC cells were transfected with the indicated plasmids for 24 hours. Subsequently, the cells were lysed in IP lysis buffer supplemented with protease and phosphatase inhibitors for 30 minutes. After centrifugation at 12,000 rpm and 4 °C, the protein supernatants were incubated overnight at 4 °C with specific antibodies and protein G agarose beads (Bestchrom, China). The beads were then washed three times with buffers containing 300 mM and 150 mM NaCl, respectively, and finally eluted with 2× SDS loading buffer prior to immunoblotting analysis. Antibody details are listed in Table S1.

Immunofluorescence and immunohistochemistry

Immunofluorescent Cells were fixed with 4% paraformaldehyde (Generalbiol, China) for 20 min at room temperature and permeabilized with 0.05% Triton X-100 (Sigma-Aldrich, USA) for 10 min. After blocking with 2% BSA in PBS for 1 h at room temperature, the samples were incubated with primary antibodies at 4°C overnight, followed by a 1h incubation with Alexa Fluor-conjugated secondary antibodies at room temperature. Nuclei were counterstained with Hoechst 33342 (Sigma-Aldrich, USA), and images were acquired using laser scanning confocal microscopy. In terms of immunohistochemistry studies, tissue sections were incubated with primary antibodies at 4°C overnight. Immune reactions were visualized the next day using 3,3′-diaminobenzidine (DAB) (Generalbiol, China) according to the manufacturer’s instructions.

ROS detection assay

Intracellular ROS levels were assessed using the Reactive Oxygen Species Detection Kit (S0033M, Beyotime, Beijing, China) following the manufacturer's instructions. Briefly, cells seeded in 6-well plates were incubated with serum-free medium containing DCFH-DA for 30 min at 37 °C in the dark. After washing with PBS, DCF fluorescence was visualized and captured using a fluorescence microscope (Zeiss, Germany), and quantitatively analyzed by flow cytometry (CytoFLEX, Beckman Coulter)

C11-BODIPY assay

For the measurement of lipid ROS levels, cells were seeded in 6-well plates and incubated with 2 μmol/L C11-BODIPY (Thermo Fisher Scientific, USA) in serum-free medium for 30 min in the dark. After staining, the cells were harvested and resuspended in 500 μL PBS. Analysis was performed using a BD Biosciences flow cytometer with excitation at 488 nm and emission at 525 nm. Lipid ROS levels were quantified based on the fluorescence intensity.

Reduced Glutathione (GSH) assay and Oxidized Glutathione (GSSG) content assay

The glutathione/oxidized glutathione (GSH/GSSG) ratio was determined using commercial assay kits (S0053, Beyotime, Beijing, China) according to the manufacturer's instructions. Cell lysates were prepared, and the levels of GSH and GSSG were quantified by measuring the absorbance at 412 nm. The GSH/GSSG ratio was then calculated based on the resulting values.

TEM

Cells were fixed first with 2.5% glutaraldehyde for 12 h at 4 °C and then with 2% osmium tetroxide. After washing, the samples were stained with 1% uranyl acetate aqueous solution; dehydrated sequentially in 50, 70, 90, 95, and 100% ethanol; and immersed in

Eponate 12 resin. The samples were then cut into ultrathin sections and counterstained with uranyl acetate and lead citrate. Finally, images were acquired using a transmission electron microscope (120 kV; Tecnai G2 Spirit, FEI).

Animal models

Subcutaneous Tumor Model

Huh7 or Hep3B cells (1×10^6 cells in 100 μL PBS) transduced with shRNAs (sh1 and sh2), Lv-DCAF4, or corresponding negative controls were subcutaneously injected into the right flank of each 6-week-old male BALB/c nude mouse (n = 6). In NAC treatment experiments, oral administration of either vehicle (distilled water) or NAC (120 mg/kg/day) was initiated seven days after tumor inoculation and continued for four weeks. For BT, a treatment planning system (TPS) was used to guide the implantation of one ^{125}I seed (37.0 MBq, 1.0 mCi) per tumor on day 7 after cell injection. Based on TPS estimates, the cumulative radiation dose delivered to 1 cm^3 of tumor tissue was projected to reach 41.36 Gy by 14 days post-implantation. At the experimental endpoint, tumors were excised, and their volumes were calculated as: volume (mm^3) = $0.5 \times width^2 \times length$.

Orthotopic Transplantation Model

Huh7 cells (3×10^6) stably expressing luciferase and transduced with shRNAs (sh1 and sh2), Lv-DCAF4, or their corresponding negative controls were injected into the livers of 6-week-old male BALB/c nude mice (n = 6). Oral administration of vehicle or NAC (120 mg/kg/day) started on day 7 and continued for four weeks. Tumor growth was monitored via bioluminescence imaging and microMRI. After imaging and euthanasia, all orthotopic tumors were excised and measured.

DEN/CCl4-Induced HCC Model

Two-week-old male C57BL/6J mice received a single intraperitoneal injection of diethylnitrosamine (DEN; 25 mg/kg). Starting two weeks later, carbon tetrachloride (CCl_4; 0.5 μL/g) was administered intraperitoneally once per week until the mice reached 26

weeks of age. In NAC-treated cohorts, mice received oral NAC (120 mg/kg/day) or vehicle from 12 weeks of age.

Reb-M@Lips-cRGD synthesis and treatment

Reb-M@Lips-cRGD were prepared by the thin-film hydration method. Briefly, lecithin (E80, 4.92 mg), cholesterol (1.25 mg), DSPE-PEG-cRGD (0.75 mg), and Reb-M (0.5 mg) were dissolved in a mixture of methanol (1 mL) and chloroform (3 mL). The organic solvents were evaporated under reduced pressure, and the resulting film was hydrated with aqueous solution. The suspension was then probe-sonicated to reduce particle size, followed by centrifuge at 13,000 rpm for 30 minutes at 4°C, and collect the pellet. Unmodified blank liposomes, used as a control, were prepared similarly but without DSPE-PEG-cRGD. For in vivo evaluation, Reb-M@Lips-cRGD was administered via tail vein injection twice-weekly.

Supplementary figure legends

Supplementary Fig. S1.

(A)TBHP dose-response curve of Huh7 cells.

(B)Log_2-transformed gRNA read count distributions.

(C)Normalized gene-level β-score for the top candidate genes identified in the CRISPR-Cas9 screen.

(D)Venn diagram of significantly enriched genes based on the MAGeCK analysis of β-score.

(E)ASPM and DCAF4 expression levels in Huh7 using qRT-PCR.

(F)Stacked bar chart showing the quantities of various cell types in tumor and paracancerous tissues.

(G) Stacked bar chart illustrating the proportions of various cell types in tumor and paracancerous tissues.

(H)Bar graph displaying the numbers of various cell types in the integrated single- cell dataset.

(I)Ro/e analysis demonstrating the tissue preference of various cell types between tumor and paracancerous tissues.

(J)Stacked bar chart presenting the relative proportions of each cell type in tumor versus paracancerous tissues.

(K)Heatmap showing the top 5 marker genes for each cell type.

(L-M) DCAF4 expression levels in HCC cell lines and human normal hepatocytes THLE-2 using qRT-PCR and western blotting.

(N)Volcano plots display the differentially expressed genes between Huh7-NC and Huh7-DCAF4-knockdown cell.

(O)Heatmap displaying differentially expressed genes (DEGs) between control and DCAF4-knockdown Huh7 cells.

(P)KEGG pathway enrichment analysis of the DEGs identified upon DCAF4 knockdown.

(Q-V)Expression levels of DCAF4 in HCC cell lines after transfection using qRT-PCR and western blotting.

*P < 0.05; **P < 0.01; ***P < 0.001.

Supplementary Fig. S2.

(A)Z-score of converted relative content of differential metabolites in Huh7 cells between the si-NC and si-DCAF4 group.

(B)Volcano plots of significantly different metabolites in Huh7 cells between the si-NC and si-DCAF4 group.

(C)KEGG pathway enrichment analysis for up-regulated and down-regulated metabolites.

(D-I)The accumulation of lipid ROS of Huh7 and HCCLM3 cells transfected with sh-NC, sh-1, or sh-2 or Hep3B cells transfected with Lv-NC or Lv-DCAF4 was detected by flow cytometry using C11-BODIPY 581/591.

(J-K)Apoptosis in HCCLM3 cells transfected with sh-NC, sh-1, or sh-2 and treated with NAC (2 mM, 24 h), analyzed by flow cytometry using an Annexin V/PI Apoptosis Detection Kit.

(L-M)Apoptosis in Hep3B cells transfected with Lv-NC or Lv-DCAF4 and treated with BT(4 Gy), analyzed by flow cytometry using an Annexin V/PI Apoptosis Detection Kit.

*P < 0.05; **P < 0.01; ***P < 0.001.

Supplementary Fig. S3.

(A-C)Nude mice injected with DCAF4-overexpressed or control Hep3B cells were administered BT, followed by a tumor formation assay.Tumor growth and weight were statistically analyzed in the subcutaneous xenograft model. Scale bars: 1 cm.

(D)IHC staining of xenograft tissues for TUNEL, DCAF4, and Ki67. Scale bars: 50 μm.

(E)Representative immunofluorescence images and quantitative analysis of DHE levels in each group. Scale bars: 50 μm.

(F)Schematic workflow of establishing the hepatocyte-specific DCAF4 knockout mice using the AAV8-TBG-Cre system.

(G)Validation of DCAF4 expression in livers after AAV8-TBG-Ctrl or AAV8-TBG-Cre administration using western blotting.

*P < 0.05; **P < 0.01; ***P < 0.001.

Supplementary Fig. S4.

(A) Cell lysates from control and DCAF4-overexpressing Hep3B cells, transfected with or without si-CUL4A, were IP with an anti-KEAP1 antibody, followed by immunoblotting (IB) with anti-Ub and anti-KEAP1 antibodies. Before harvesting, cells were treated with 20 μM MG132.

(B)KEAP1 protein levels in control and DCAF4-overexpressing Hep3B cells, transfected with or without si-CUL4A.

(C)Relative mRNA levels of HO1 and NQO1 in control and DCAF4-overexpressing Hep3B cells, transfected with or without si-CUL4A.

(D)Analysis of proteomic databases indicates that 2 lysine residues of KEAP1 are potentially ubiquitinated.

(E)Sequence alignment of the conserved K323 and K551 containing region in KEAP1 orthologs of different species.

(F)Sequence alignment of the conserved K131 and K312 containing region in KEAP1 orthologs of different species.

*P < 0.05; **P < 0.01; ***P < 0.001.

Supplementary Fig. S5.

(A-B)Flow cytometric analysis (left) and quantification (right) of Huh7 cells transfected with sh-NC, sh-1, or sh-2, showing intracellular ROS levels via DCFH-DA staining after 24 h treatment with 5 μM ML385.

(C-D)Representative images (left) and quantification (right) of colony formation assays in DCAF4-overexpressing Hep3B cells treated with ML385 (5 μM).

(E)Immunofluorescence analysis of G3BP1 and DCAF4 in Huh7 cells treated with BT. Scale bars: 10 μm.

(F-G)Immunofluorescence analysis of G3BP1 and KEAP1 in HCCLM3 cells transfected with sh-NC or sh-DCAF4 and treated with BT. Line-scan profiles reflect fluorescence intensities along the indicated path. Scale bars: 10 μm.

(H)Western blot analysis of KEAP1 in HCC cells transfected with si-NC or si-G3BP1 after BT.

(I-J)Co-IP analysis of the DCAF4-KEAP1 interaction in HCC cells transfected with si-NC or si-G3BP1 after BT.

(K-L)Nuclear fractionation and western blot analysis of NRF2 in HCC cells transfected with si-NC or si-G3BP1 after BT

(M-N) Flow cytometry and quantification of intracellular ROS levels assessed by DCFH-DA staining in DCAF4-knockdown HCC cells treated with ISRIB (200 nM) under BT.

(O-R)ROS levels measured by DCFH-DA staining and apoptosis analysis in HCC cells transfected with si-NC or si-G3BP1 after BT.

*P < 0.05; **P < 0.01; ***P < 0.001.

Supplementary Fig. S6.

(A) Predicted transcription factors for DCAF4 analyzed by TF-Target Finder.

(B-C) RT-qPCR analysis determined the relative mRNA expression levels of DCAF4 in Huh7 and HCCLM3 cells under TBHP-induced oxidative stress at 0, 6, 12, and 24 hours.
(D-E) Western blot analysis revealed a consistent upregulation of DCAF4 protein expression in both Huh7 and HCCLM3 cells following TBHP-induced oxidative stress at 0, 6, 12, and 24 hours.

(F-I) siRNA-mediated knockdown of XBP1 in oxidative stress-induced Huh7 and HCCLM3 cells led to a significant reduction in both DCAF4 mRNA and protein levels.

(J) CUT&Tag-seq profiling confirmed the enrichment of XBP1 binding peaks at the DCAF4 genomic locus.

(K) Putative XBP1-binding sites were identified within the promoter region of the DCAF4 gene.

(L) Dual-luciferase reporter assays demonstrated that mutation in Region 1 abolished the ability of XBP1 to activate the DCAF4 promoter.

(M)ChIP assays further validated the binding of XBP1 to Region 1.

(N) Representative multiplex immunohistochemistry images illustrated the staining patterns of XBP1 and DCAF4 in human hepatocellular carcinoma (HCC) tissues. Scale bar = 200 μm.

(O) A positive correlation between XBP1 and DCAF4 expression was observed in 98 clinical HCC specimens.

(P) Analysis of the GEPIA database also indicated a significant correlation between XBP1 and DCAF4 mRNA expression levels in HCC.

$^{*}P < 0.05$, $^{**}P < 0.01$, $^{***}P < 0.001$.

Supplementary Fig. S7.

(A)Chemical structure of Reb-M.

(B)The encapsulation efficiency and loading capacity of Reb-M in Lips-cRGD.

(C)The in vitro release profile of Reb-M in PBS buffer at 37°C.

(D)IHC staining of xenograft tissues for TUNEL and Ki67. Scale bars: 50 μm.

(E)The body weight was measured and recorded every 3 days.

(F-G)AST and ALT levels in blood plasma of each group.

(H)H&E staining of histological sections from major organs, including heart, liver, spleen, lung and kidney, scale bar, 50μm.

*P < 0.05; **P < 0.01; ***P < 0.001.

Supplementary Fig. S8.

(A)The respective cell clusters were defined based on specific markers for the respective cell type.

(B)The TSNE plot visualized the distribution of cell clusters in Ctrl and Reb-M@Lips-cRGD group.

(C-F)TSNE plot showing the distribution of specific markers.

Supplementary Table 1: Antibodies used in this study

Antibodies	Source	Cat no.
anti-DCAF4	ThermoFisher	PA5-61485
	Novus Biologicals	NBP1-05972
	proteintech	31440-1-AP
anti-KEAP1	proteintech	10503-2-AP
anti-GAPDH	proteintech	10494-1-AP
anti-Lamin B1	proteintech	12987-1-AP
anti-NRF2	proteintech	16396-1-AP
anti-acetylated-lysine	Cell Signaling Technology	9441
anti-CUL4A	proteintech	14851-1-AP
anti-G3BP1	abcam	ab181150
	proteintech	66486-1-1g
anti-Flag	proteintech	20543-1-AP
anti-Myc	proteintech	60003-2-Ig
anti-Tubulin	proteintech	11224-1-AP
anti-XBP1	proteintech	24868-1-AP
anti-Ub	proteintech	10201-2-AP

Supplementary Table 2: Primers sequences used for real-time PCR

Gene	Species	Primer Sequence(5'-3')
DCAF4	Human	TTGCCATTGATCTGCGTTGTG<br>GTACTGCCTTACGCACTTCG
HO-1	Human	AGTCTTCGCCCCTGTCTACT<br>CTTCACATAGCGCTGCATGG
NQO-1	Human	AACACTGCCCTCTTGTGGTG<br>GCTCGGTCCAATCCCTTCAT
GAPDH	Human	TCCAAAATCAAGTGGGGCGA<br>AAATGAGCCCCAGCCTTCTC
XBP1	Human	TGACATCCAGCAGTCCAAGG<br>GCAAGCCAGGATGCCAAAAA

Supplementary Table 3: shRNA and siRNAs used in this study

Gene	Species	Sequence(5'-3')
shDCAF4-1	Human	GGGACCTGAGGACCACGAAGT
shDCAF4-2	Human	GCATCACTGTTCGTCAATAGT
shDCAF4-3	Human	GGACCAACAGTGATGTCTTGG
siDCAF4	Human	CCUGAAUAUCCAAGCAAAUTT
siASPM	Human	UCUGAAACACCAAGCUAUAUUTT
siXBP1	Human	GCCAAGCUGGAAGCCAUUAAUTT
siG3BP1	Human	CAUUAACAGUGGUGGGAAATT
siCUL4A	Human	ACUGUUUAGAACCCAUAUUAUTT

Supplementary Table 4: Correlations between DCAF4 in patients with HCC (n=98)

Characteristics	Number	DCAF4 expression		P-value
		Low	High	
Age (years)				
<60	51	29	22	0.157
≥60	47	20	27	
Gender				
Female	14	10	4	0.0833
Male	84	39	45	
Cirrhosis				
Present	48	22	26	0.4189
Absent	50	27	23	
HBV infection				
Positive	69	31	38	0.1214
Negative	29	18	11	
Tumor size (cm)				
<5	52	34	18	**0.0012**
≥5	46	15	31	
Microvascular invasion				
Presence	31	10	21	**0.0169**
Absence	67	39	28	
Tumor multiplicity				
Single	72	33	39	0.1698
Multiple	26	16	10	
α-fetoprotein (ng/ml)				
≤200	57	33	24	0.0653
>200	41	16	25	
Edmondson stage				
I/II	47	30	17	**0.0086**
III/IV	51	19	32	
